# Aromatic amino acid biosynthesis impacts root hair development and symbiotic associations in *Lotus japonicus*

**DOI:** 10.1093/plphys/kiad398

**Published:** 2023-07-10

**Authors:** Jesús Montiel, Ivette García-Soto, Euan K James, Dugald Reid, Luis Cárdenas, Selene Napsucialy-Mendivil, Shaun Ferguson, Joseph G Dubrovsky, Jens Stougaard

**Affiliations:** Departamento de Genómica Funcional de Eucariotas. Centro de Ciencias Genómicas, Universidad Nacional Autónoma de México, Cuernavaca 62210, Mexico; Department of Molecular Biology and Genetics, Aarhus University, Aarhus DK-8000, Denmark; Departamento de Genómica Funcional de Eucariotas. Centro de Ciencias Genómicas, Universidad Nacional Autónoma de México, Cuernavaca 62210, Mexico; Ecological Sciences, The James Hutton Institute, Invergowrie, Dundee DD2 5DA, UK; Department of Molecular Biology and Genetics, Aarhus University, Aarhus DK-8000, Denmark; Department of Animal, Plant and Soil Sciences, School of Agriculture, Biomedicine and Environment, La Trobe University, Melbourne, Victoria 3086, Australia; Departamento de Biología Molecular de Plantas, Instituto de Biotecnología, Universidad Nacional Autónoma de México, Cuernavaca 62210, Mexico; Departamento de Biología Molecular de Plantas, Instituto de Biotecnología, Universidad Nacional Autónoma de México, Cuernavaca 62210, Mexico; Department of Molecular Biology and Genetics, Aarhus University, Aarhus DK-8000, Denmark; Departamento de Biología Molecular de Plantas, Instituto de Biotecnología, Universidad Nacional Autónoma de México, Cuernavaca 62210, Mexico; Department of Molecular Biology and Genetics, Aarhus University, Aarhus DK-8000, Denmark

## Abstract

Legume roots can be symbiotically colonized by arbuscular mycorrhizal (AM) fungi and nitrogen-fixing bacteria. In *Lotus japonicus*, the latter occurs intracellularly by the cognate rhizobial partner *Mesorhizobium loti* or intercellularly with the *Agrobacterium pusense* strain IRBG74. Although these symbiotic programs show distinctive cellular and transcriptome signatures, some molecular components are shared. In this study, we demonstrate that 3-deoxy-d-arabino-heptulosonate 7-phosphate synthase 1 (DAHPS1), the first enzyme in the biosynthetic pathway of aromatic amino acids (AAAs), plays a critical role in root hair development and for AM and rhizobial symbioses in *Lotus*. Two homozygous *DAHPS1* mutants (*dahps1-1* and *dahps1-2*) showed drastic alterations in root hair morphology, associated with alterations in cell wall dynamics and a progressive disruption of the actin cytoskeleton. The altered root hair structure was prevented by pharmacological and genetic complementation. *dahps1-1* and *dahps1-2* showed significant reductions in rhizobial infection (intracellular and intercellular) and nodule organogenesis and a delay in AM colonization. RNAseq analysis of *dahps1-2* roots suggested that these phenotypes are associated with downregulation of several cell wall–related genes, and with an attenuated signaling response. Interestingly, the *dahps1* mutants showed no detectable pleiotropic effects, suggesting a more selective recruitment of this gene in certain biological processes. This work provides robust evidence linking AAA metabolism to root hair development and successful symbiotic associations.

## Teaser

The regulation of aromatic amino acid biosynthesis in the legume *Lotus japonicus* is crucial for sustaining root hair growth and establishing mutualistic relationships with beneficial microbes.

## Introduction

Legume roots establish interactions with beneficial microorganisms in the rhizosphere through complex chemical dialogs. Arbuscular mycorrhizal symbiosis (AMS) and root nodule symbiosis (RNS) are 2 well-known examples, which provide phosphorus and nitrogen sources, respectively, to the host (Oldroyd 2013). In the model legume *Lotus* (*Lotus japonicus*), 2 modalities of root colonization exist in RNS, depending on the microorganism partner (Montiel et al. 2021; Quilbé et al. 2022). The cognate rhizobial partner *Mesorhizobium loti* synthesizes and releases signaling molecules called lipochito-oligosaccharide nodulation factors (NFs), after perception of flavonoid compounds secreted by roots (Bek et al. 2010; Shimamura et al. 2022). Recognition of these compounds activates the symbiotic signaling pathway that allows rhizobial infection and nodule development. *M. loti* attaches to the root hair tip, inducing it to curl, thereby trapping the bacteria within infection pockets, where a tubular structure called an infection thread (IT) is formed. In this process, the rhizobial partner colonizes the root cell layers intracellularly, through root hair ITs, which advance toward the cortex, where a nodule primordium, generated by reactivation of the cell division in the root cortex, is formed. The bacteria are released from the ITs into the nodule cells as organelle-like structures, called symbiosomes, differentiating into nitrogen-fixing bacteroids (Roy et al. 2020). However, it is estimated that approximately 25% of all legume genera employ an alternative modality of rhizobial colonization, called intercellular infection (Sprent 2007). Our group recently showed that *Lotus* can also be infected intercellularly by the *Agrobacterium pusense* strain IRBG74 that was originally isolated from the flooding-tolerant legume *Sesbania cannabina* (Cummings et al. 2009; Mitra et al. 2016). In this entry mode, IRBG74 induces massive root hair curling and twisting but no root hair ITs are formed; instead, the bacteria pass between the epidermal root cells, forming cortical infection pockets. The migration proceeds both intra- and intercellularly into the nodule cells, releasing the bacteria from transcellular ITs or intercellular infection structures (Montiel et al. 2021). In the *Lotus*–IRBG74 symbiosis, root hair ITs are not formed but the bacteria are attached to the root hairs, which probably favors the nodulation program.

Strigolactones secreted by the roots are perceived by AM fungi, promoting spore germination and hyphal branching. These responses favor the physical interaction of the fungal hyphae with the root epidermal cells, giving rise to the formation of the hyphopodium (Bonfante and Genre 2010). The fungal hyphae penetrate the epidermal cells, reaching the cortex, where they differentiate into branched hyphae called arbuscules, where carbon and phosphorous sources are exchanged with the plant host (MacLean et al. 2017). Molecular genetic studies have identified important players involved in the signaling pathway that allow the mutualistic associations. In *Lotus*, crucial components of the symbiotic route comprise plasma membrane receptors, transcription factors, and a range of proteins that orchestrate the colonization of rhizobia through the root cell layers (Roy et al. 2020). The intercellular colonization of IRBG74 in *Lotus* roots shows evident cellular and transcriptome differences, with distinct genetic requirements with respect to the root hair IT formation process. However, there is a core of symbiotic genes that are indispensable for any modality of rhizobial infection (Montiel et al. 2021; Quilbé et al. 2022), which belong to the common symbiotic signaling pathway (CSSP) and also play essential roles in AMS (Stracke et al. 2002; Lévy et al. 2004; Parniske 2008; Yano et al. 2008).

The root hairs are extensions of specialized epidermal cells with polarized growth and a tubular shape, which increase the surface area for nutrient acquisition. In Arabidopsis (*Arabidopsis thaliana*), the characterization of a large collection of mutants affected during root hair development and emergence indicates that these processes are regulated by a sophisticated network that encompasses various molecular functions (Shibata and Sugimoto 2019). Root hairs also play a crucial role during RNS, in the early signaling pathway and in the intracellular colonization of rhizobia (Downie 2014). Actin cytoskeleton rearrangements and cell wall modifications in the root hairs are necessary to facilitate the formation and progression of ITs (Cárdenas et al. 2003; Yokota et al. 2009; Hossain et al. 2012; Zepeda et al. 2014; Qiu et al. 2015; Su et al. 2023). Several regulators of root hair development also participate in the establishment of symbiotic associations with rhizobia and AM fungi (Karas et al. 2005; Lei et al. 2015; Ke et al. 2016; Liu et al. 2020; Karas et al. 2021).

The functioning of the nitrogen-fixing nodule and arbuscules is regulated by a large array of metabolic genes. In these symbiotic organs, several enzymes of diverse metabolic pathways modulate the flux of carbon, phosphorous, and nitrogen sources between the root cells and the microsymbionts (Udvardi and Poole 2013). However, transcriptome analyses of legume roots inoculated with compatible rhizobia and AM fungi show that reprograming of metabolic processes also occurs at early stages of the symbiotic associations (Manthey et al. 2004; Deguchi et al. 2007; Handa et al. 2015; Fonseca-García et al. 2021). Despite the evident activation of several metabolic routes during the colonization and organogenesis programs in the mutualistic interactions of legumes, their role has been poorly explored. In this study, we found that expression of a *Lotus 3-deoxy-d-arabino-heptulosonate 7-phosphate synthase* (*LjDAHPS*) gene was associated with several stages of RNS and root hair development. *DAHPS1* encodes the first enzyme of the shikimate pathway, which leads to the biosynthesis of the aromatic amino acids (AAAs) phenylalanine, tyrosine, and tryptophan (Lemaître et al. 2014). These amino acids serve as precursors of various metabolites, including phytohormones and cell wall components (Tzin and Galili 2010; Geng et al. 2020; Simpson et al. 2021). The characterization of 2 homozygous mutant alleles disrupted in *LjDAHPS1* revealed a pivotal role of this gene in root hair development and in the establishment of symbiotic associations with nitrogen-fixing bacteria and AM fungi.

## Results

### A *DAHPS* is induced during the *Lotus*–IRBG74 symbiosis

**Figure 1.**
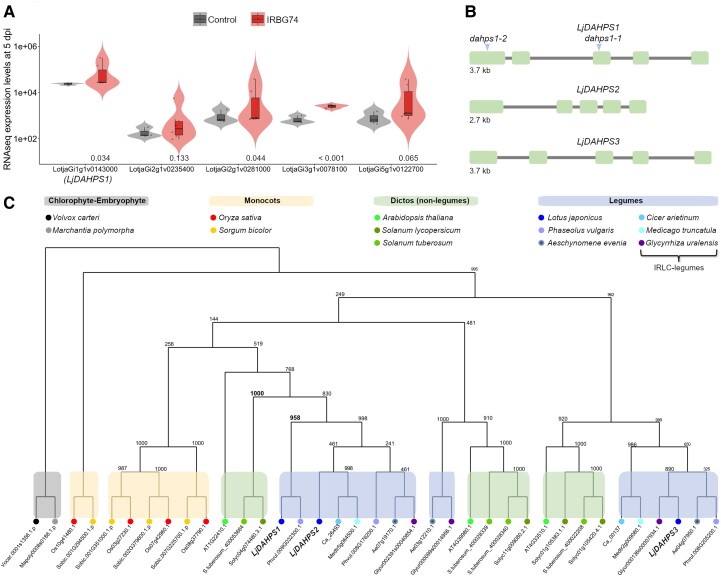
Prominent expression of *LjDAHPS1* during *Lotus*–IRBG74 symbiosis, gene structure, and phylogeny of DAHPS in various plants. **A)***DAHPS1* was the most abundant transcript among the upregulated genes in Gifu roots at 5 dpi with IRBG74. The violin boxplots were generated with data calculated from RNAseq information (Montiel et al. 2021). Violin boxplots: center line, median; box limits, upper and lower quartiles; whiskers, 1.5× interquartile range; points, individual data points. The *P*-adjust values are indicated below the violin boxplots. **B)** Graphic representation of the exon (green box) and intron (gray line) composition in the *LjDAHPS1* (LotjaGi1gv0143000), *LjDAHPS2* (LotjaGi1g1v0239000), and *LjDAHPS3* (LotjaGi6g1v0351200) genes. Blue arrowheads indicate the retrotransposon insertions in the *dahps1*-1 (30141487) and *dahps1*-2 (30100225) alleles. **C)** Phylogenetic relationship of DAHPS in various legumes and nonlegumes (monocots and dicots). The chlorophyte (*Volvox carteri*) and embryophyte (*Marchantia polymorpha*) DAHPS were included as roots. Bootstrap values are shown at the nodes.

We have recently shown that IRBG74 induces a distinctive transcriptome response during the intercellular infection of *Lotus* roots (Montiel et al. 2021). Among the regulated genes, LotjaGi1g1v0143000 encoding a 3-deoxy-d-arabino-heptulosonate 7-phosphate synthase (hereafter referred as *DAHPS1*) was the most abundant transcript (Fig. 1A and Supplemental Table S1). DAHPS1 is the first enzyme of the shikimate pathway (Supplemental Fig. S1A) involved in the biosynthesis of AAAs (Tzin and Galili 2010), which suggests an unexpected role for amino acid homeostasis in RNS. Supporting this notion, *DAHPS1* is highly expressed in NF-treated root hairs and nodule primordia (Supplemental Fig. S1C). A further investigation of the gene in the *Lotus* database (https://lotus.au.dk/) shows that *DAHPS1* contains 5 exons and 4 introns (Fig. 1B). To determine whether *DAHPS1* belongs to a gene family, a BLAST was performed in the *Lotus* genome (Kamal et al. 2020). Two homologous genes were identified, LotjaGi1g1v0239000 and LotjaGi6g1v0351200, which were named *LjDAHPS2* and *LjDAHPS3*, respectively (Fig. 1B). Unlike *LjDAHPS1*, *LjDAHPS2* and *LjDAHPS3* were not evidently induced by IRBG74 at any timepoint studied (Supplemental Fig. S1B) and showed lower expression levels in root hairs, nodule primordia, and mycorrhized roots (Supplemental Fig. S1C). The deduced amino acid sequence of *DAHPS1* encodes a protein of 539 amino acids, with a predicted plastid signal peptide (Supplemental Fig. S2). Similarly, DAHPS2 and DAHPS3 contain an N-terminal plastid-targeting sequence. The protein sequence of these 3 *Lotus* enzymes is highly conserved (Supplemental Fig. S2), sharing >80% identities. To investigate the phylogeny of *Lotus* DAHPS, a phylogenetic tree was constructed using full-length protein sequences (Supplemental Table S2). In this analysis, the *Lotus* DAHPS proteins were compared with homologous sequences found in the legumes *Aeschynomene evenia*, chickpea (*Cicer arietinum*), liquorice (*Glycyrrhiza uralensis*), *Medicago truncatula*, and common bean (*Phaseolus vulgaris*). In addition, their phylogenetic distribution was evaluated with their counterparts in several nonlegumes (monocots and dicots) with fully sequenced genomes, i.e. rice (*Oryza sativa*), sorghum (*Sorghum bicolor*), *A. thaliana*, tomato (*Solanum lycopersicum*), and potato (*Solanum tuberosum*). The DAHPS family was composed of 4 members in the monocots *O. sativa* and *S. bicolor*, while in the dicots, it ranged between 2 and 4 members (Fig. 1C). Particularly, legumes of the inverted repeat-lacking clade (IRLC) contained only 2 DAHPS homologs, except for *G. uralensis* that possesses 3 (Fig. 1C). The different DAHPSs clustered in subclades that reflect the phylogeny of the species.

### *DAHPS1* promoter activity in different root tissues and during RNS

**Figure 2.**
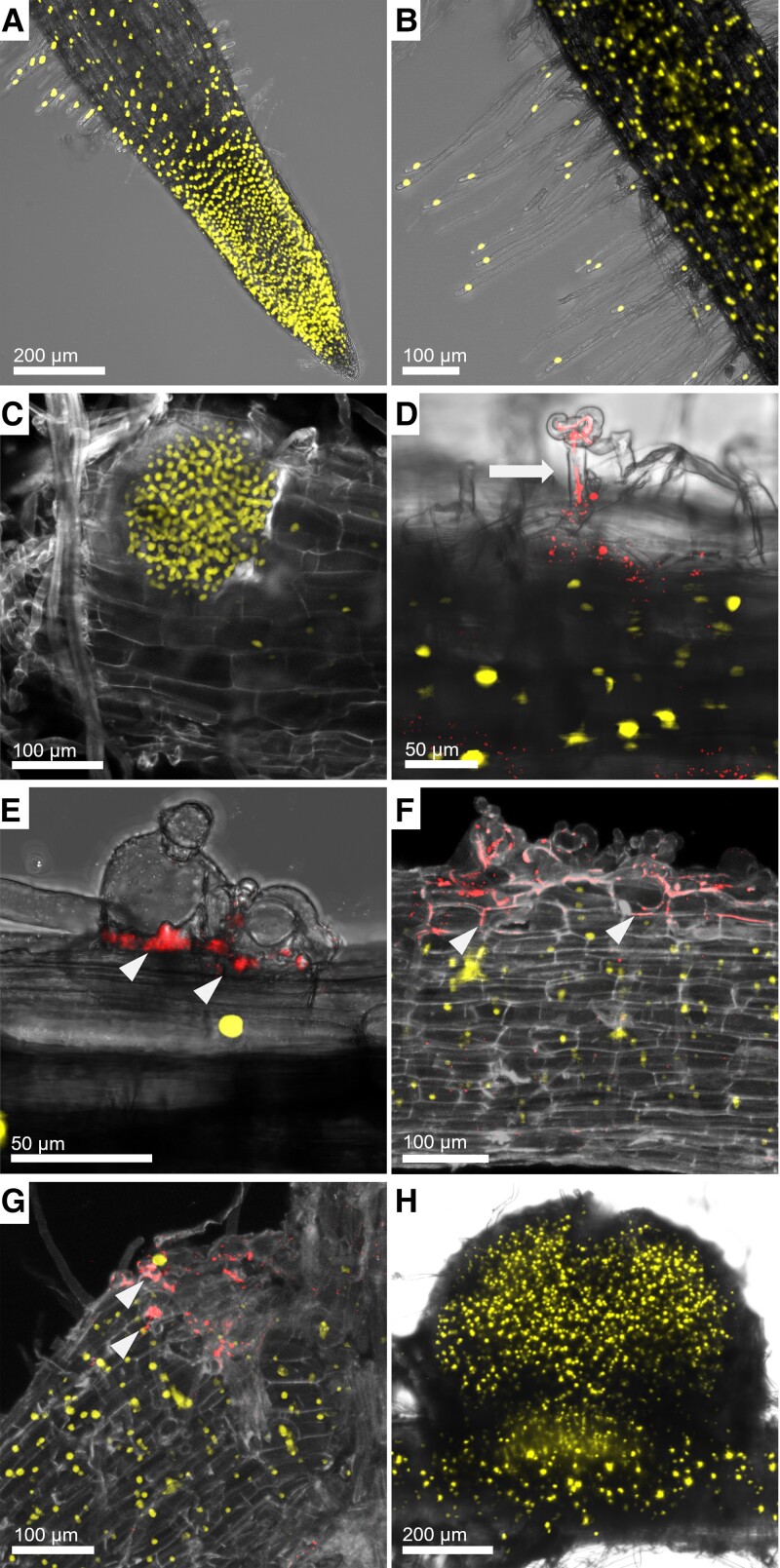
Detection of *DAHPS1* promoter activity in various *Lotus* root tissues during RNS. *DAHPS1* promoter activity was visualized by confocal microscopy in uninoculated (**A to C**) and inoculated (**D to F**) *Lotus* transgenic roots of comparable developmental stages harboring the *pDAHPS1::tYFP-nls* construct (2 kb of the *DAHPS1* promoter sequence fused to the triple YFP with a nuclear localization signal). *tYFP-nls* was clearly expressed at the root tip **A**), root hairs **B**), and emerging lateral root primordium **C**). Strong promoter activity in cells surrounding the root hair IT **D**), the intercellular infection **E)**, and nodule organogenesis at 1 wpi with *M. loti*–DsRed **D**) and 2 wpi with IRBG74–DsRed **E to H)**. Arrow and arrowheads indicate the IT and intercellular infection, respectively. Scale, 50 *µ*m **D, E**), 100 *µ*m **B, C**), and 200 *µ*m **A, F**).

Transcriptome profile sources indicate that *DAHPS1* is expressed in roots and potentially plays a role in RNS of *Lotus*. To explore the cellular expression pattern, we monitored the activity of the *DAHPS1* 2-kb promoter sequence fused to the triple yellow fluorescent protein (YFP) with a nuclear localization signal (*pDAHPS1::tYFP-nls*). Strong expression of the fluorescent reporter was detected in the apical region of the root, growing root hairs, and emerging lateral root primordia of uninoculated roots, comprising different root tissues (Fig. 2, A to C). Likewise, after *M. loti*–DsRed and IRBG74–DsRed inoculation, *pDAHPS1::tYFP-nls* was clearly detected in the epidermal and cortical cells adjacent to intracellular and intercellular infection sites, respectively (Fig. 2, D to F). An elevated promoter activity remained during intercellular colonization and nodule organogenesis (Fig. 2, G and H), which indicates the participation of *DAHPS1* during nodule development.

### *LjDAHPS1* is dispensable for shoot and root growth but crucial during root hair development

**Figure 3.**
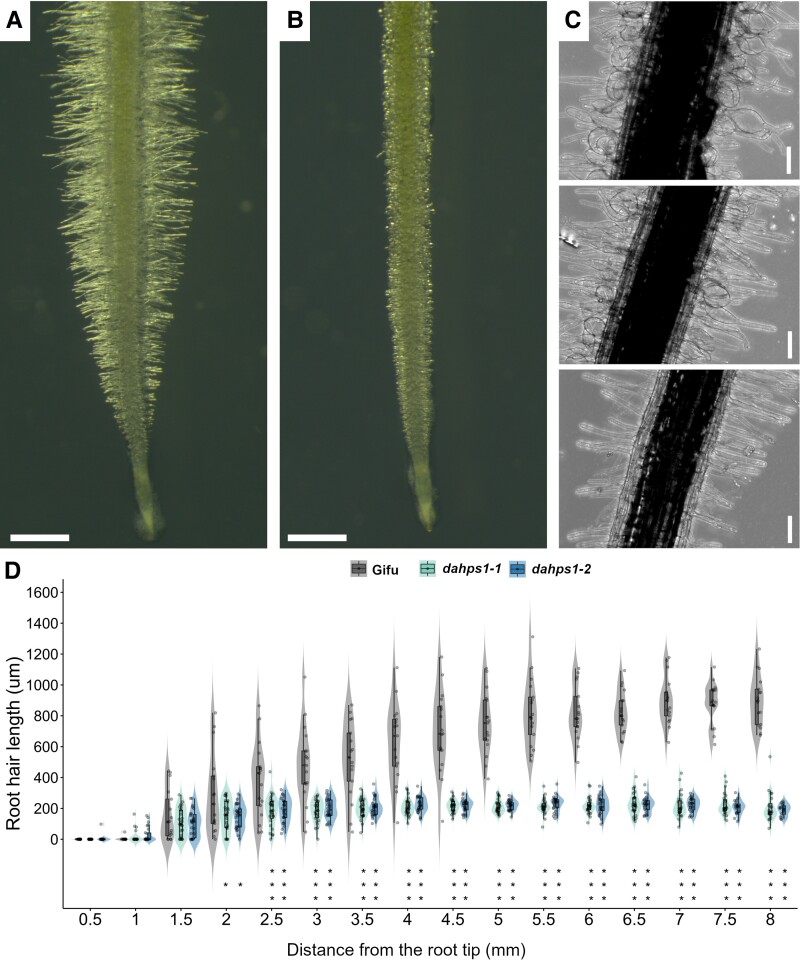
Altered morphology and growth profile of root hairs in the *dahps1*-1 and *dahps1*-2 mutants. Representative images of the roots in Gifu **A)** and the *dahps1*-1 mutant **B)** (the same phenotype was observed for the *dahps1*-2 mutant) at 10 dpg. **C)** Sequential images of the progressive deterioration of root hair structure along the root axis of a *dahps1*-1 mutant at 10 dpg (bottom to top). Scale 1 mm **A**, **B)** and 100 *µ*m **C)**. **D)** Violin boxplots of the maximal root hair length recorded at different distances from the root tip in Gifu, *dahps1*-1, and *dahps1*-2 roots at 10 dpg. Student's *t*-test of root hair length between Gifu (*n* = 17) and the *dahps1*-1 (*n* = 27) and *dahps1*-2 (*n* = 25) mutants. *P < 0.05; ***P < 0.001. Violin boxplots: center line, median; box limits, upper and lower quartiles; whiskers, 1.5× interquartile range; points, individual data points.

To further investigate the potential role of *DAHPS1* in the symbiotic associations of *Lotus*, 2 homozygous mutant alleles, *dahps1*-1 and *dahps1*-2, were obtained from the LORE1 mutant collection (Urbanski et al. 2012; Małolepszy et al. 2016) and these contained retrotransposon insertions in the third and first exon, respectively (Fig. 1B). The evidence obtained on the expression profile of *DAHPS1* led us to first explore the root phenotype in the *dahps1*-1 and *dahps1*-2 mutants. Both the primary root length and the root apical meristem (RAM) length of plants at 10 d post germination (dpg) were not significantly different in the 2 mutant alleles, when compared to those of wild-type Gifu (hereafter referred as Gifu) plants (Supplemental Fig. S3, A to C). Similarly, the root growth dynamics in the *dahps1*-1 and *dahps1*-2 mutants was comparable to that recorded for Gifu roots, at least during a period of 1 to 11 dpg (Supplemental Fig. S3D). Likewise, no significant differences were detected in the shoot length in Gifu and *dahps1* plants at 5 wk post germination (wpg), grown in nitrogen-replete conditions (Supplemental Fig. S3, E and F). However, the root hair development was dramatically altered in the *dahps1*-1 and *dahps1*-2 mutants, compared to Gifu (Fig. 3, A and B). Although the root hairs seem to emerge normally in the differentiation zone of the *dahps1*-1 and *dahps1*-2 mutants with a typical tubular shape, they underwent a progressive swelling, while developing and aging, forming balloon-like structures that eventually collapsed (Fig. 3C). Unlike the root hairs of Gifu plants that reached up to 1 mm in length, the root hairs of *dahps1*-1 and *dahps1*-2 mutants barely reached 400 *µ*m, reflecting the drastic impact of their abnormal development (Fig. 3, A to D). Considering that the mutant and wild-type roots grew at similar growth rates and that changes in root hair length began to be detected at 1.5 to 2 mm from the root tip (Fig. 3D), the data suggest that root hair apical growth in the mutants was arrested soon after root hair bulge formation. This phenotype is consistent with the results obtained on the promoter activity of *DAHPS1*, linking the regulation of AAA biosynthesis to root hair development.

### Actin cytoskeleton and cell wall integrity are compromised in root hairs of the *DAHPS1* mutants

**Figure 4.**
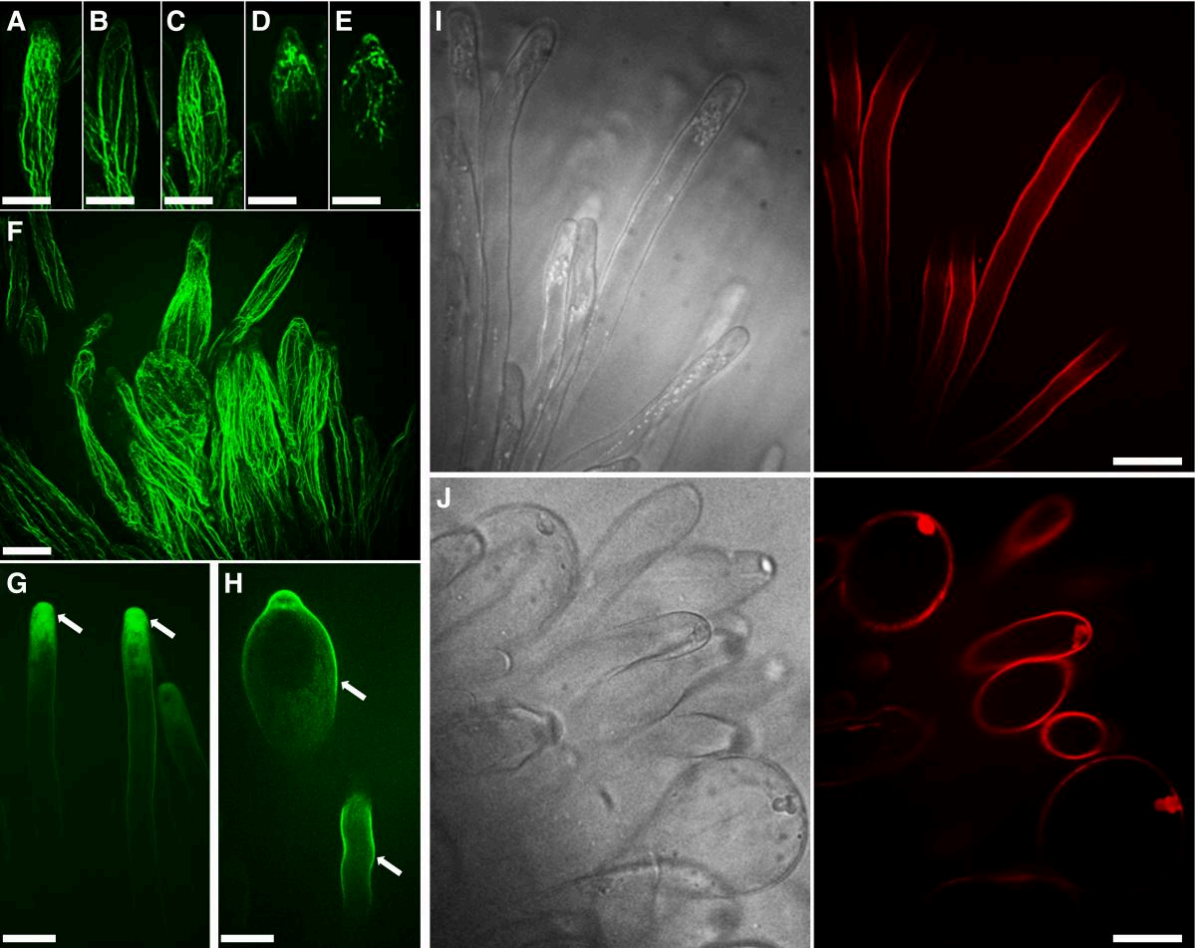
Disruption of actin cytoskeleton organization and cell wall dynamics in the root hairs of *dahps1* mutants. Imaging of the actin microfilament organization in Gifu **A)**, *dahps1*-1 **B to E)**, and *dahps1*-2 **F)** root hairs stained with Alexa-Phalloidin at 5 dpg. Visualization of the actin polymerization sites in living root hairs of Gifu **G)** and the *dahps1*-1 **H)** mutant, with fluorescently labeled cytochalasin D at 5 dpg. The arrows indicate the polymerization sites at the root tip and subapical region of root hairs in Gifu and *dahps1*-1, respectively. Visualization of demethoxylated pectins at the cell wall of Gifu **I)** and *dahps1*-2 **J)** root hairs by PI staining at 5 dpg. Bright field and fluorescence images are shown in the left and right panels, respectively. Scale, 20 *µ*m.

The drastic alteration in the root hair morphology of the *dahps1*-1 and *dahps1*-2 mutants prompted us to study this phenomenon in more detail by analyzing their actin cytoskeleton dynamics. The actin microfilaments, visualized by Alexa-Phalloidin staining, formed long bundles parallel to the long axis of Gifu root hairs (Fig. 4A). Similarly, these structures were observed in the root hairs of the *dahps1*-1 and *dahps1*-2 mutants with an early and mild swelling (Fig. 4, B, C, and F). However, the progressive alterations in the root hair morphology were accompanied by a gradual disruption of the actin cytoskeleton (Fig. 4, D to F). A similar scenario was detected in the epidermal and cortical cells, where a sophisticated network of long and abundant bundles was formed in Gifu, while in the *dahps1* mutants, the actin cytoskeleton was composed of fewer and short bundles (Supplemental Fig. S4, A to C). To further understand the dramatic changes in the cytoskeleton organization, the actin polymerization sites were monitored in growing root hairs incubated with fluorescently labeled cytochalasin D (Cyt-Fl). In Gifu root hairs, the major fluorescent signal was detected in the apical region, reflecting the fast-growing (plus) ends of microfilaments (Fig. 4G). In deformed root hairs of the *dahps1*-1 and *dahps1*-2 mutants, the fluorescence was considerably reduced in the apical region, and interestingly, sites of actin polymerization were associated with subapical regions (Fig. 4H). This displacement of actin polymerization sites seems to be linked to the misorientation of actin bundles (Fig. 4F) and would explain the root hair phenotype.

The root hair growth and morphology is also regulated by changes in the cell wall dynamics. To assess the composition of the root hair cell wall in the *dahps1* mutants, the roots were incubated with propidium iodide (PI), a fluorescent dye that binds the demethoxylated pectin in the hardened cell walls of living root hairs (Rounds et al. 2011). In Gifu, the PI fluorescence was predominantly associated with the root hair shanks, but almost absent at the tip, where the cell wall is more extensible (Fig. 4I). By contrast, in the *dahps1*-2 mutant, the dye was detected in the periphery of the swollen root hairs (Fig. 4J) and even localized within intracellular structures (Fig. 4J). Our results suggest that the progressive loss of normal root hair morphology in the *dahps1*-2 mutants is caused by perturbations in the growth dynamics and organization of actin microfilaments, along with changes in the distribution of cell wall components.

### Pharmacological and genetic restoration of root hair morphology

*DAHPS1* encodes the first enzyme in the biosynthetic pathway of chorismate, a precursor of AAAs. To evaluate its predicted enzymatic function, a heterologous complementation approach was conducted. The *Escherichia coli* mutant strain NT1402 (Jayaraman et al. 2022), which lacks all 3 cognate DAHPS enzymes, was transformed with the *LjDAHPS1* coding sequence (CDS). NT1402 and the complemented strains, NT1402-LjDAHPS1_1 and NT1402-LjDAHPS1_2, grew very well in media supplemented with shikimate and AAAs with casamino acids, which contains a mixture of essential amino acids except for tryptophan (Supplemental Fig. S5A). In the absence of casamino acids, the growth of the different mutants was drastically perturbed, but in the plates where phenylalanine and tyrosine were present without tryptophan, only microcolonies developed in the NT1402 mutant, while in the complemented mutants, the growth was substantially increased (Supplemental Fig. S5B). This result supports the predicted role of *LjDAHPS1* as a relevant component in the AAA biosynthetic pathway.

**Figure 5.**
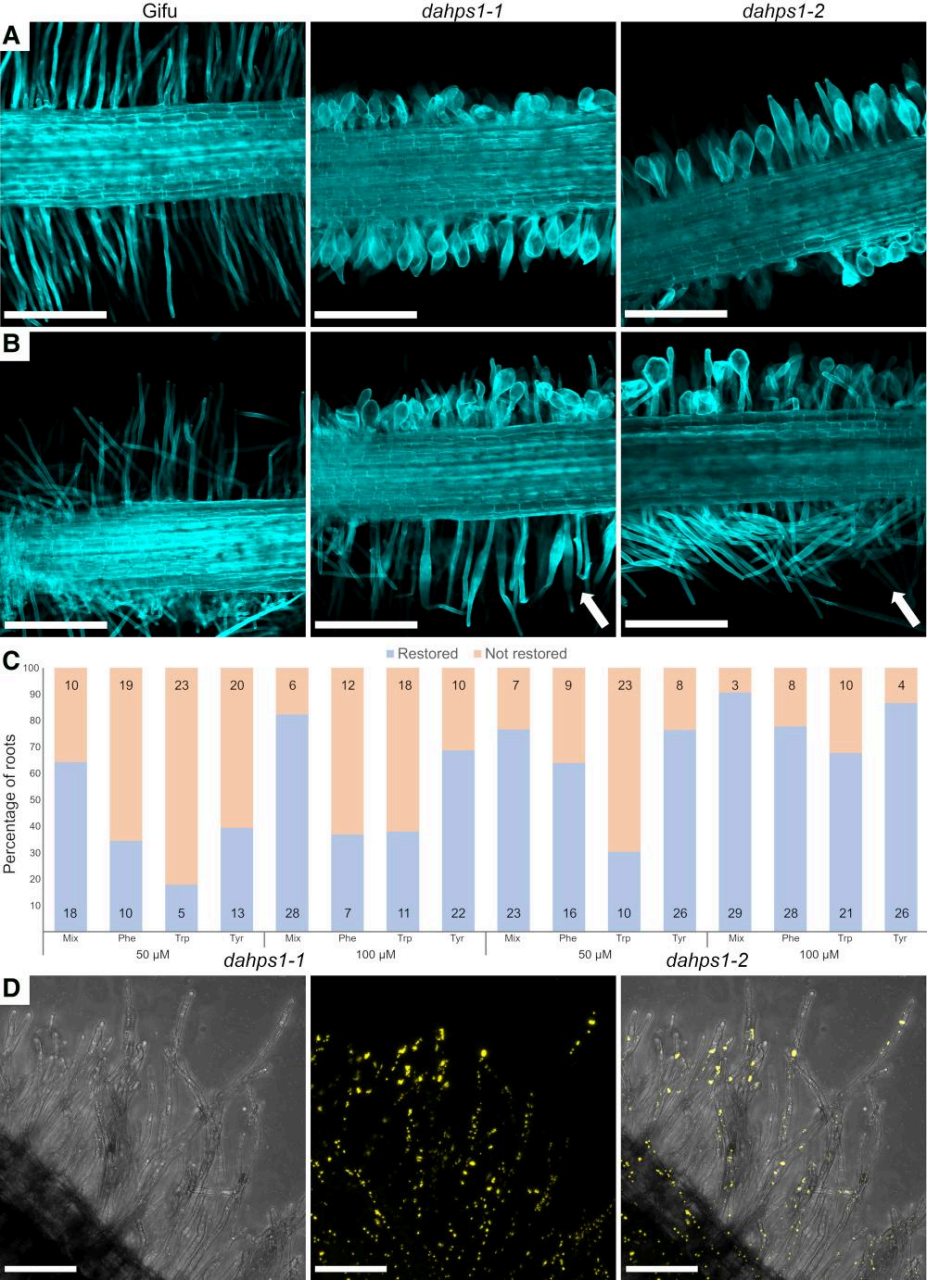
Restoration of the root hair tubular shape in the *dahps1*-1 and *dahps1*-2 mutants. Confocal microscopy images of Gifu, *dahps1*-1, and *dahps1*-2 roots grown in MS medium **A**) supplemented with an equimolar combination of Phe, Trp, and Tyr 100 *µ*M **B)** at 10 dpg. The tubular root hair morphology was restored only in the root tissue that was in contact with the agar (indicated with arrows). Scale, 500 *µ*m. **C)** proportion of plants with restored root hair morphology (tubular shape). The number of plants tested is indicated within the bars. **D)** Long root hairs with tubular shape observed by confocal microscopy in *dahps1*-2 transgenic roots transformed with the *pDAHPS1::DAHPS1-YFP* construct. Left panel, transmitted light; middle panel, yellow fluorescence; right panel, merged images. Scale, 100 *µ*m.

To corroborate if the altered root hair morphology and growth in the *dahps1*-1 and *dahps1*-2 plants were linked to a deficiency of AAA levels, the root hair development was analyzed in 10 dpg seedlings of the different genotypes, germinated and grown on MS medium supplemented with an equimolar concentration of phenylalanine, tryptophan, and tyrosine. Root hairs with a restored tubular shape were observed in the 2 mutant alleles grown with a 50 and 100 *µ*M mixture of AAAs (Fig. 5, A and B, and Supplemental Fig. S6A). Interestingly, this effect was only present in the root tissue that was in contact with the agar that contained the cocktail of amino acids (Fig. 5B). To elucidate whether phenylalanine, tryptophan, or tyrosine was separately contributing to prevent the altered root hair morphology and growth in the *dahps1*-1 and *dahps1*-2 mutants, the individual amino acids were added to the growth medium at 50 and 100 *µ*M. The root hair swelling was prevented in both mutant alleles grown in medium supplemented with each amino acid, although to different extents (Supplemental Fig. S6A). The percentage of plants with restored tubular root hair structure was 68% to 86% with tyrosine, 36% to 77% with phenylalanine, and 38% to 61% with tryptophan (Fig. 5C). As described earlier, besides the swelling, the apical root hair growth is also arrested in the *DAHPS1* mutants. In this regard, the maximal root hair length in the *adl1*-1 and *dahps1*-2 mutants was not restored to the levels in Gifu by adding different combinations and concentrations of AAAs to the medium (Supplemental Fig. S6B). This result suggests that the root hair structure and growth are independently impacted by AAAs. Interestingly, the apical root hair growth of Gifu plants grown on media supplemented with AAAs was also affected (Supplemental Fig. S6, A and B), reinforcing the notion that apical root hair growth is influenced by AAAs.

Using a chemical approach, we found that an imbalance in AAA levels was linked to the root hair phenotype in the *dahps1*-1 and *dahps1*-2 mutants. To demonstrate that this deficiency was caused by the disruption of the *LjDAHPS1* gene, we made a construct composed of its CDS fused to the YFP transcriptionally regulated by the native promoter (*pDAHPS1::DAHPS1-YFP*). The root hair morphology and development were restored in *dahps1*-2 transgenic roots expressing the *pDAHPS1::DAHPS1-YFP* construct (Fig. 5D). Additionally, a clear fluorescent signal of the reporter was detected throughout root hair development (Supplemental Fig. S6C), supporting our previous findings. The fluorescence was associated with plastid-like structures that match with the presence of a plastid signal peptide in the predicted amino acid sequence of *DAHPS1*.

### Gene expression network associated with *DAHPS1*

The evidence collected in this study demonstrates that disruption of *DAHPS1*, a gene involved in the biosynthetic pathway of AAAs, has drastic consequences on root hair development. The data suggest that an insufficiency of these amino acids likely impacts the expression profile of genes and biological processes intrinsically connected to these molecules. We addressed this hypothesis by analyzing the transcriptome of *dahps1*-2 roots by RNAseq in 5-dpg seedlings. Compared with the root expression profile in Gifu plants of the same age, a total of 416 differentially expressed genes (DEGs; *P*-adjust < 0.5 and Log2FC ≥ 2) were detected (Supplemental Fig. S7A and Table S3). From this list, 163 sequences were upregulated and 253 were repressed in *dahps1*-2. Nineteen downregulated genes were related to the regulation of cell wall biomechanics. Interestingly, the Cystathionine-β-Synthase-like1 (*CBS*) and *Vapyrin* genes, crucial for rhizobial infection in *M. truncatula* (Murray et al. 2011; Sinharoy et al. 2016), were upregulated in the *dahps1*-2 roots compared to Gifu (Supplemental Fig. S7B). Similarly, the expression of *Nodule inception* (*NIN*), a master transcriptional regulator of rhizobial colonization and nodule organogenesis (Schauser et al. 1999), was induced in the uninoculated *dahps1*-2 roots (Supplemental Fig. S7B).

**Figure 6.**
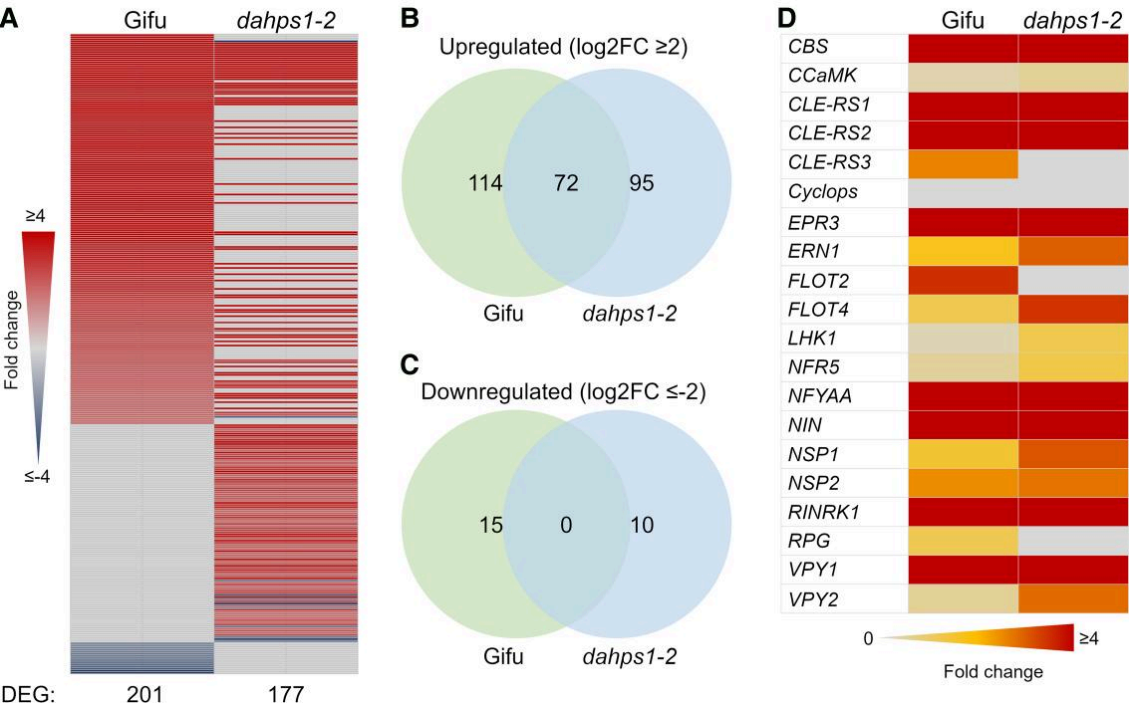
Attenuated gene expression response in *dahps1*-2 roots after IRBG74 inoculation. Heatmap **A)** and Venn diagrams **B, C)** show a reduced number of DEGs in *dahps1*-2 roots compared to Gifu at 5 dpi with IRBG74, but several early symbiotic genes were similarly induced **D)** (*P*-adjust < 0.5). The values were obtained from RNAseq analysis and are calculated with respect to uninoculated roots of equivalent age on the same genotype.

We previously described that *DAHPS1* was induced in Gifu roots at 5 dpi with IRBG74, when the major transcriptome response is observed in the intercellular infection (DEG Log2FC ≥ 2). To explore the contribution of *DAHPS 1* to the symbiotic signaling in the *Lotus*-IRBG74 interaction, the expression pattern of *dahps1*-2 roots was analyzed at 5 dpi with IRBG74. Using low stringency parameters (*P*-adjust < 0.5), we detected 2,840 DEGs in the inoculated Gifu roots and only 1,475 in *dahps1*-2 (Supplemental Fig. S7C and Table S4). However, the number of DEGs with Log2FC ≥ 2 was somewhat similar, 177 and 201 in *dahps1*-2 and Gifu, respectively (Fig. 6A). Interestingly, less than 40% of these sequences overlapped (72) and none of the downregulated genes overlapped (Fig. 6, B and C). Despite the low similarity in the expression profiles between Gifu and *dahps1*-2 roots (Fig. 6, A to C), a core set of early symbiotic genes displayed comparable expression levels after IRBG74 inoculation (Fig. 6D). This set of genes encodes receptors (Exopolysaccharide Receptor 3 [EPR3]; Lotus Histidine Kinase [LHK1]; Nod Factor Receptor 5 [NFR5]; and Rhizobial Infection Receptor-Like Kinase1 [RINRK1]), transcriptional regulators (Nuclear-Localized Coiled-Coil Protein [CYCLOPS]; ERF Required for Nodulation [ERN1]; Nuclear Factor Y [NFYA]; Nodule Inception [NIN]; Nodulation-Signaling Pathway [NSP1]; and Nodulation-Signaling Pathway [NSP2]), and proteins of diverse functions that regulate rhizobial colonization and nodule organogenesis (Cystathionine-β-Synthase-Like [CBS]; Calcium/Calmodulin-Dependent Protein Kinase [CCaMK]; Clavata3/Embryo Surrounding Region [CLE-RS1, CLE-RS2, andCLE-RS3]; Flotillin [FLOT2, FLOT4]; Rhizobium-directed Polar Growth [RPG]; and Vapyrin [VPY1, VPY2]) (Roy et al. 2020).

### *DAHPS1* contributes to different modalities of rhizobial infection and nodule organogenesis

**Figure 7.**
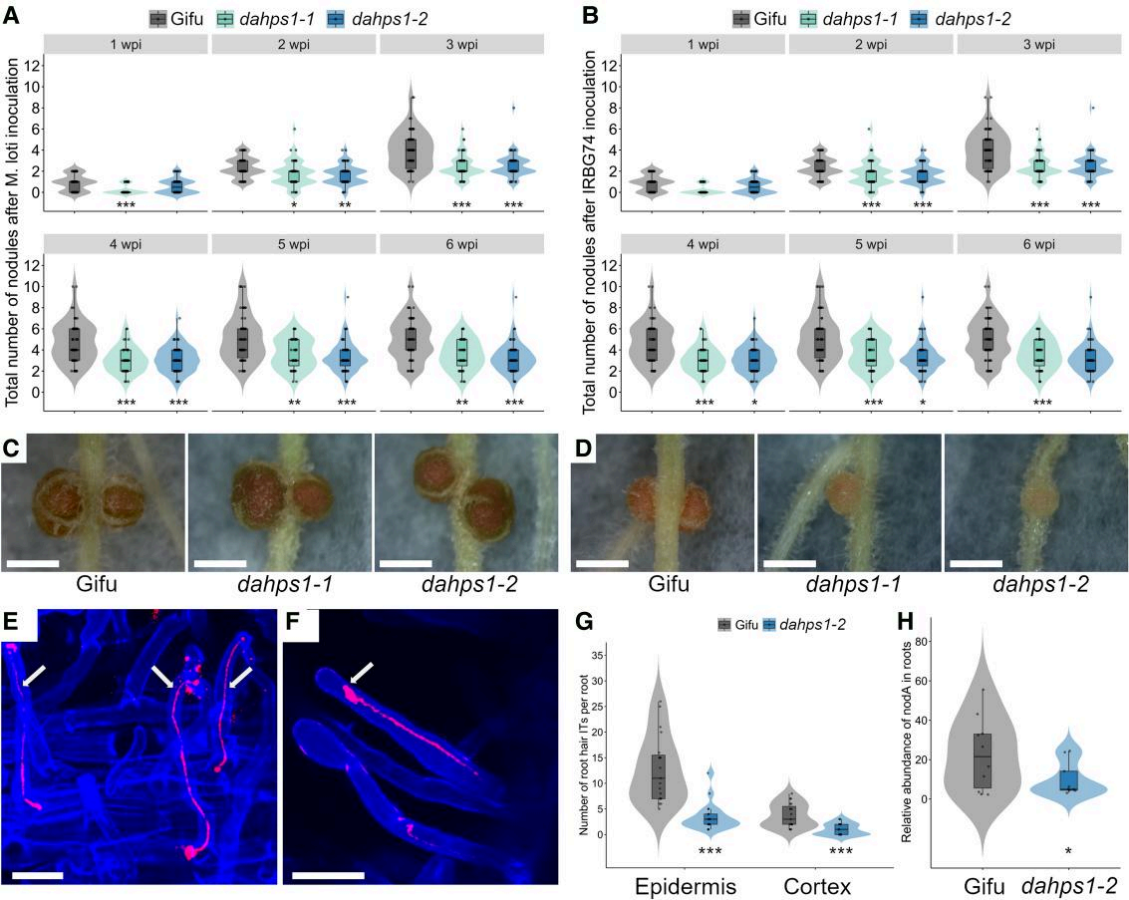
Nodulation phenotype of *dahps1*-1 and *dahps1*-2 mutants after *M. loti* and IRBG74 inoculation. Total number of nodules recorded on Gifu (*n* ≥ 29), *dahps1*-1 (*n* ≥ 43), and *dahps1*-2 (*n* ≥ 29) at 1 to 6 wpi with *M. loti***A)** and IRBG74 **B)**. Mann–Whitney *U* test of the total number of nodules (asterisks below the violin graphs indicate significant difference: *P < 0.05; **P < 0.01; ***P< 0.001) between Gifu and the *DAHPS1* mutant alleles. Representative images of nodules formed on different *Lotus* genotypes at 3 wpi with *M. loti***C)** and IRBG74 **D)**. Scale, 1 mm. Root hair infection in Gifu **E)** and *dahps1*-2 **F)** visualized by confocal microscopy at 1 wpi with *M. loti*–DsRed. The arrows indicate the ITs. Scale bar, 20 *µ*m. **G)** Number of root hair ITs found in the epidermis and cortex at 1 wpi with *M. loti*–DsRed on Gifu (*n* = 19) and *dahps1*-2 (*n* = 19). **H)** Abundance of IRBG74-nodA by qPCR in genomic DNA isolated from Gifu (*n* = 10) and *dahps1*-2 (*n* = 9) roots at 3 wpi with IRBG74 and normalized to the LotjaGi1g1v0152000 gene accumulation. Student's *t*-test of *nodA* abundance between roots of Gifu and the mutants. *P < 0.05 and ***P < 0.001. Violin boxplots: center line, median; box limits, upper and lower quartiles; whiskers, 1.5× interquartile range; points, individual data points.

The RNAseq analysis in the *dahps1*-2 roots inoculated with IRBG74 indicates that disruption of *LjDAHPS1* affects the symbiotic transcriptome response. To evaluate the relevance of this finding, a nodulation kinetics analysis was conducted on plates for the *dahps1*-1 and *dahps1*-2 mutants, recording the number of pink nodules and the total number of nodules at 1 to 6 wpi with *M. loti* and IRBG74. Nodule formation (pink and total) was significantly reduced in both mutant alleles at most of the timepoints analyzed, compared to Gifu (Fig. 7, A to D, and Supplemental Fig. S8, A and B). The delayed and reduced nodulation of *dahps1*-1 and *dahps1*-2 mutants had a negative influence on plant growth, since the length of the aerial parts was significantly shorter compared to that of Gifu at 6 wpi with any of the inocula used (Supplemental Fig. S8, C and D). These findings reveal that *DAHPS1* makes an important contribution to the nodulation capacity of *Lotus*. Since the root hair and nodulation phenotypes were similar in the 2 mutant alleles, we decided to perform a detailed symbiotic characterization only for the *dahps1*-2 allele.

First, the intracellular invasion of *M. loti*-DsRed was monitored on Gifu and *dahps1*-2 roots by confocal microscopy. Root hair ITs were observed both in Gifu and in *dahps1*-2 at 1 wpi. In the latter, the infection events only occurred in root hairs with mild morphological alterations; however, the typical root hair curling was not detected; instead, the IT initiation took place in a subapical region (Fig. 7, E and F). Moreover, at this timepoint, the number of epidermal and cortical ITs *per* root was significantly lower in the *dahps1*-2 mutant compared to Gifu (Fig. 7G), which might be an indirect consequence of the defect in root hair development. Since the intercellular infection of IRBG74 in *Lotus* is technically unsuitable for a quantitative evaluation through microscopy techniques, we followed a root endosphere approach; the *NodA* gene abundance was estimated by qPCR in DNA samples extracted from *Lotus* roots at 3 wpi with IRBG74 (Montiel et al. 2021). This analysis indicated that *dahps1*-2 roots had a significant reduction of approximately 50% in the relative abundance of the IRBG74-*nodA* gene, compared to Gifu (Fig. 7H). These approaches revealed that both intra- and intercellular infections were negatively impacted in the *dahps1*-2 mutant; however, the effect on the intracellular colonization was more dramatic, with a 70% reduction.

**Figure 8.**
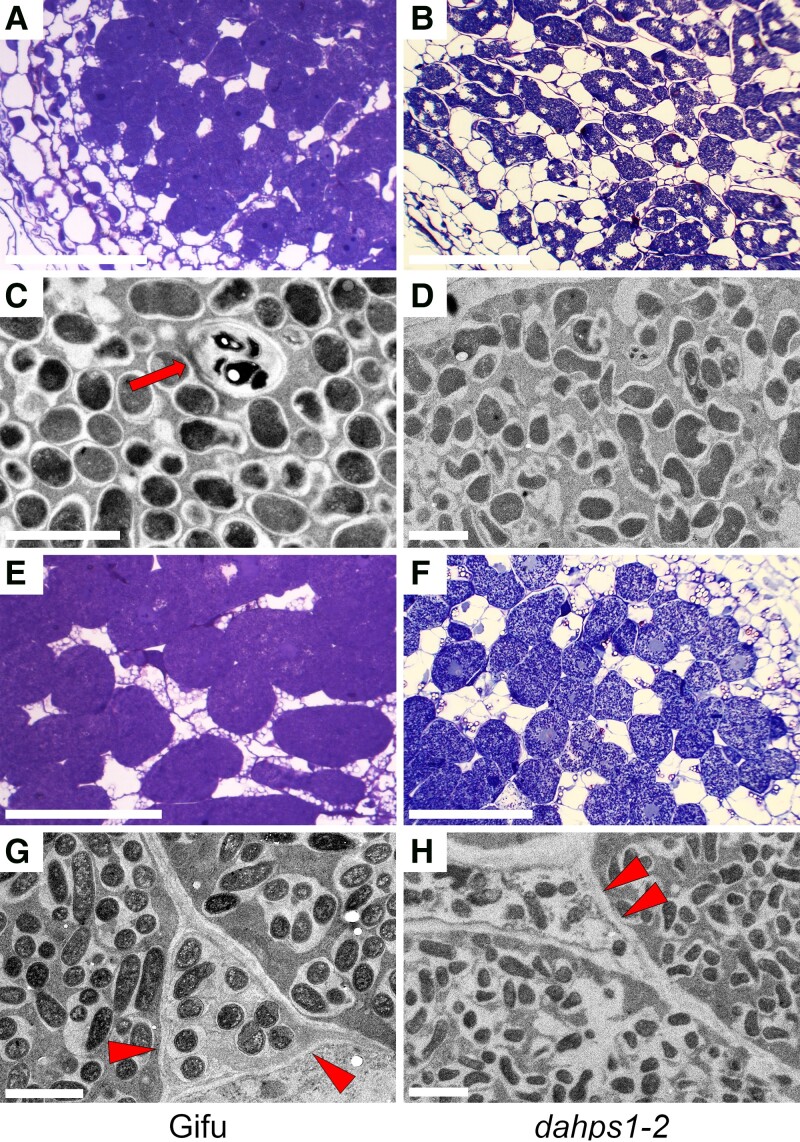
Reduced bacteroid occupancy and altered symbiosome structure in nodules formed in the *dahps1*-2 mutant. Representative images of nodule sections and symbiosomes at 3 wpi with *M. loti***A, C, E, and G**) and IRBG74 **B, D, F, and H)** obtained by transmitted light **A, B, E, and F)** and TEM **C, D, G, and H)** in Gifu (left panels) and *dahps1*-2 (right panels). Arrow and arrowheads indicate transcellular ITs and peg- infection, respectively. Scale, 2 *µ*m **C, D, G, and H)** and 50 *µ*m **A, C, E, and G)**.

Taken together, these results show that an efficient intra- and intercellular symbiotic infection in *Lotus* depends on the function of *DAHPS1*. These findings prompted us to investigate the bacteroid colonization in the nodules formed by *M. loti* and IRBG74 in the *dahps1*-2 mutant. Infected nodule cells were detected in histological slides, stained with toluidine blue, of 3-wk-old nodules inoculated with *M. loti* and IRBG74 in Gifu (Fig. 8, A and E) and *dahps1*-2 (Fig. 8, B and F). In Gifu nodules, the infected cells were densely packed with *M. loti* (Fig. 8A) and IRBG74 (Fig. 8E) bacteroids. By contrast, the infected cells in *dahps1*-2 nodules were vacuolized with both inocula, showing a deficient filling of the cells by bacteroids (Fig. 8, B and F). Based on these findings, we decided to investigate the symbiosome structure by transmission electron microscopy (TEM). In Gifu nodules colonized by *M. loti*, the infected cells contained transcellular ITs with round-shaped symbiosomes hosting 1 to 2 bacteroids (Fig. 8C). Although the nodules in *dahps1*-2 also contained 1 or 2 *M. loti* bacteroids per symbiosome, these organelle-like structures had irregular shapes (Fig. 8D). These abnormalities were also detected in the *dahps1*-2 nodules formed by IRBG74, which were accompanied by an evident disruption in the space of the symbiosome (Fig. 8H), contrasting with the integrity of this structure observed in Gifu (Fig. 8G). Altogether, these analyses thus showed that *DAHPS1* contributes to rhizobial infection and nodule organogenesis.

### AM colonization is delayed in *dahps1*-2 roots

**Figure 9.**
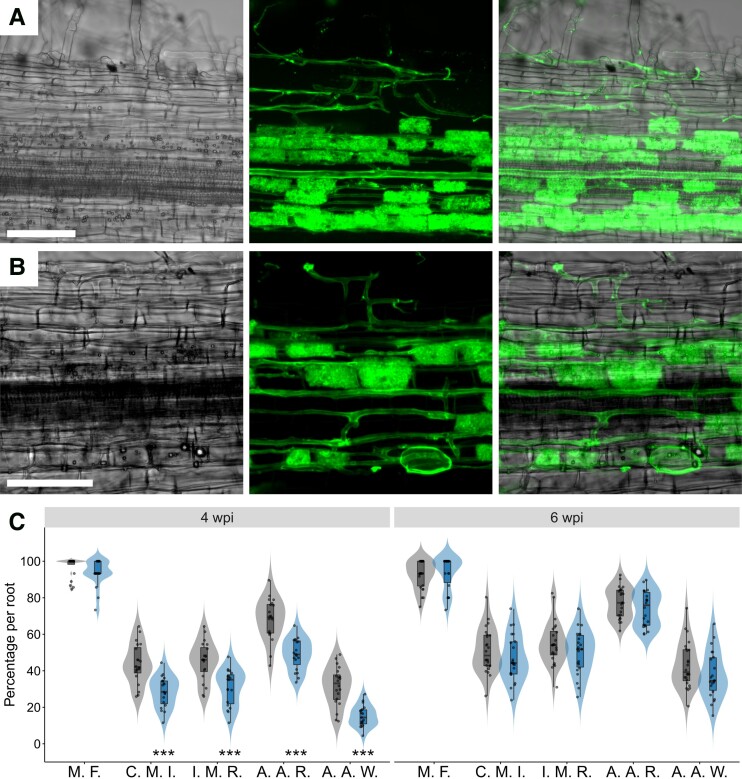
Arbuscular mycorrhization of the *dahps1*-2 mutant. Visualization by confocal microscopy of AM colonization at 4 wpi in Gifu **A)** and *dahps1*-2 **B)** root fragments stained with WGA-Alexa Fluor 488. Left panel, transmitted light; middle panel, green fluorescence; right panel, merged images. Scale, 100 *µ*m. **C)** Mycorrhizal frequency (M. F.), cortex mycorrhizal intensity (C. M. I.), intensity of mycorrhiza in the root fragments (I. M. R.), arbuscule abundance in mycorrhizal parts of root fragments (A. A. R.), and arbuscule abundance in the root system (A. A. W.) of Gifu (*n* ≥ 20) and *dahps1*-2 (*n* ≥ 19) plants at 4 and 6 wpi with *R. intraradices*. Violin boxplots: center line, median; box limits, upper and lower quartiles; whiskers, 1.5× interquartile range; points, individual data points.

The expression profile of *DAHPS1* during RNS reflects its relevant role in this mutualistic association. Likewise, the data collected from the *L. japonicus* gene expression atlas (LjGEA) show that *DAHPS1* is highly expressed in mycorrhized roots (Supplemental Fig. S1B). This prompted us to evaluate the AM phenotype of *dahps1*-2. For this purpose, 5-dpg seedlings of Gifu and *dahps1*-2 were inoculated with *Rhizophagus intraradices* spores in Magenta boxes. Root fragments of Gifu and *dahps1*-2 roots were collected at 4 wpi and stained with WGA-Alexa Fluor to visualize AM colonization by confocal microscopy. Fully branched hyphae with arbuscules within the cortical cells were observed in both genotypes, but the rate of arbuscule formation was apparently lower in the *dahps1*-2 mutant compared to Gifu (Fig. 9, A and B). Consequently, we conducted a quantitative analysis of different fungal colonization structures by optical microscopy in root segments stained with Trypan blue at 4 and 6 wpi (Trouvelot et al. 1986). Gifu and *dahps1*-2 roots showed a similar percentage of mycorrhizal frequency at 4 and 6 wpi, a parameter that reflects the presence of AM fungi in the roots (Fig. 9C). However, other indicators that reveal the degree of fungal colonization were significantly reduced at 4 wpi in *dahps1*-2: cortex mycorrhizal intensity, intensity of mycorrhiza in the root fragments, arbuscule abundance in mycorrhizal parts of root fragments, and arbuscule abundance in the root system (Fig. 9C). These results demonstrate a delay in the establishment of AMS in *dahps1*-2 plants, since the values obtained for all these parameters were similar to those in Gifu at 6 wpi (Fig. 9C). These results suggest that *DAHPS1* is required at the early stages of AM root colonization.

## Discussion

### Root hair development is AAA dependent

Root hair biology is relevant due to the role played by these tubular extensions of epidermal cells on nutrient acquisition and its particularly polarized growth. The study of mutants defective in root hair emergence and growth shows that this process is highly complex and dynamic, involving transcription factors, phytohormones, cytoskeleton rearrangement, cell wall modifications, and secondary messengers (Shibata and Sugimoto 2019). Our study demonstrates that perturbance of AAA biosynthesis in the *dahps1*-1 and *dahps1-*2 mutants influences the mechanism of root hair development. The observed progressive alteration in the root hair morphology of *dahps1*-1 and *dahps1*-2 mutants was reverted by genetic complementation with the *DAHPS1* sequence, the first enzyme in the biosynthesis of AAAs.

Besides the genetic complementation, we demonstrated that root hair swelling in *dahps1-*1 and *dahps1*-2 can be partially restored in 80% to 90% of the plants by adding a mixture of AAAs in a dose-dependent manner. This result indicates that in the *DAHPS1* mutant alleles, the root hair phenotype was caused by the lack or deficiency of these aromatic molecules or their synthesis, and not by the absence of the intermediate compounds produced in the shikimate pathway. Interestingly, the tubular shape of root hairs was recovered in 68% to 86% of the mutants grown in a medium supplemented only with 100 *µ*M of tyrosine. This finding suggests that the root hair swelling in the *dahps1*-1 and *dahps1*-2 mutants was mostly provoked by the absence of tyrosine or its derived compounds. Phosphorylation of tyrosine residues occurs in actin-related proteins (Guillen et al. 1999), affecting the cytoskeleton dynamics in diverse biological processes such as plant bending and pollen tube growth (Kameyama et al. 2000; Zi et al. 2007). We observed that both root hair morphology and the actin cytoskeleton progressively deteriorated in *dahps1*-1 and *dahps1*-2. These effects were apparently preceded by changes in the distribution of F-actin plus ends. In healthy growing root hairs, the F-actin plus ends are localized at the root tip, paving the way for its growth (Zepeda et al. 2014). By contrast, these structures were detected in subapical regions of the root hairs in the *dahps1*-1 and *dahps1*-2 mutants. The critical role played by the actin cytoskeleton during root hair development has been further supported by genetic evidence. The maintenance of the tip growth is affected in the root hairs of the *Arabidopsis* mutant *deformed root hairs 1*, affected in the major actin of the vegetative tissue (Ringli et al. 2002). In *Lotus*, the actin cytoskeleton is severely compromised in the *actin-related protein component* (*arpc1*), *nap1* (for Nck-associated protein 1), and *pir1* (for 121F-specific p53 inducible RNA) mutants (Hossain et al. 2012). Our data, along with previous reports, indicate that tyrosine has a strong influence on the cytoskeletal dynamics of plant cells and that the interference of its metabolism could impact the cell architecture.

The wide bulbous swelling of root hairs in the *dahps1*-1 and *dahps1*-2 mutants resembles the phenotype of the *root hair deficient 1* mutant in Arabidopsis, disrupted in the UDP-D-glucose 4-epimerase (UGE) enzyme, necessary for the galactosylation of cell wall components (Seifert et al. 2002). Cell wall composition and flexibility are certainly crucial to sustain the root hair shape and growth, since the list of cell wall–related mutants with defective root hairs includes the *leucine-rich repeat extensins Atlrx1* and *Atlrx2* (Baumberger et al. 2003), and the *cellulose synthases-like D* (Wang et al. 2001; Karas et al. 2021). L-Phenylalanine and L-tyrosine are products of the shikimate pathway and also serve as precursors for phenylpropanoid metabolism. These secondary metabolites include flavonoids and cell wall–associated phenolics (Vogt 2010). It was recently shown that flavonols modulate the reactive oxygen species (ROS) levels that drive root hair development in *A. thaliana*. Mutants affected in synthesis of flavonols exhibit a greater frequency of trichoblast cells forming root hairs and raised epidermal ROS levels (Gayomba and Muday 2020). Our RNAseq analysis of *dahps1*-2 roots suggests that the root hair phenotype in *dahps1*-2 could be related to a misregulation of several genes related to cell wall biomechanics such as *Expansin*, *Pectate lyase*, *Xyloglucan endoglucanase*, *CASP-like protein*, *Endoglucanase*, and *Pectinesterase*. This hypothesis is further supported by the perturbance observed in the cell wall dynamics of *dahps1*-2 root hairs stained with PI, a fluorescent dye that binds to demethoxylated pectins (Rounds et al. 2011). The absence of PI labeling at the root tip of growing root hairs is presumably related to greater extensibility properties, allowing the polar root hair growth (Rounds et al. 2011). Interestingly, the *dahps1*-2 root hairs lacked this fluorescent pattern and the PI was rather detected on the entire periphery, which likely reflects a reduced extensibility of the cell wall that prevents apical root hair growth.

### *DAHPS1*, a relevant player at different stages of the *Lotus*–rhizobia symbiosis

Evidence collected by comprehensive approaches revealed the participation of DAHPS1 at different stages of the *Lotus*–rhizobia symbiosis, through the interplay with various biological processes. *DAHPS1* expression is clearly linked to nodule formation, since its promoter was strongly activated in developing nodules and the number of these structures was significantly reduced in the *dahps1*-1 and *dahps1*-2 mutants after *M. loti* or IRBG74 inoculation. This symbiotic phenotype is probably caused by insufficient levels of AAAs and their derived compounds, in a high-demanding organogenesis program (Mergaert et al. 2020). *DAHPS1* is apparently an integral component of the genetic program governing developmental processes, since its promoter was also highly active in the RAM and emerging lateral roots. In *M. truncatula*, it was recently shown that a large proportion of the transcriptome changes in lateral root primordia also occur in developing nodules (Schiessl et al. 2019). Additionally, the delay in the nodulation kinetics observed in the *DAHPS1* mutant alleles could be provoked by deficient reprograming of the transcriptome. The RNAseq analysis of *dahps1*-2 roots inoculated with IRBG74 showed that, although the major symbiotic genes were induced, the total number of DEGs was considerably lower compared to the transcriptome response in Gifu wild type and only a minor proportion of DEGs overlapped between these 2 genotypes. *DAHPS1* is the major 3-deoxy-d-arabino-heptulosonate 7-phosphate synthase isoform expressed in *Lotus* roots; therefore, it is likely that disruption of this gene has a negative impact on the flavonoid levels derived from phenylalanine. Flavonoids are essential compounds produced by legume roots in the rhizosphere for their chemical crosstalk with rhizobia (Liu and Murray 2016), and the silencing of flavonoid biosynthesis components inhibits nodulation in soybean (*Glycine max*) and *M. truncatula* transgenic roots (Subramanian et al. 2006; Wasson et al. 2006).

In addition, disruption of *DAHPS1* also interferes with rhizobial colonization both intra- and intercellularly. The presence of IRBG74 was reduced by 50% in *dahps1*-2 roots compared to Gifu, at 3 wpi. However, the impact on the intracellular infection by *M. loti* was more dramatic, as the number of epidermal and cortical root hair ITs diminished by >70% in *dahps1*-2 at 1 wpi compared to Gifu. Additionally, root hair curling, a key structure to trap rhizobia and initiate IT formation, was not observed in *dahps1*-2 plants. The compromised rhizobial colonization is likely influenced by the abnormal and collapsed root hairs in the *DAHPS1* mutants, caused by the perturbed cytoskeletal organization in the root hairs. Cytoskeleton rearrangements are necessary for the root hair rhizobial infection (Yokota et al. 2009; Hossain et al. 2012; Qiu et al. 2015), and apparently, the intercellular invasion also depends on this response, since *Lotus* mutants disrupted in cytoskeleton-related genes show a severe nodulation phenotype with IRBG74 (Copeland 2021; Montiel et al. 2021).

The analysis of legume mutants indicates that the genetic requirements for RNS and AMS are partially overlapping, leading to the concept of a common symbiosis signaling pathway (CSSP) (Parniske 2008). Recent phylogenomic studies confirm that certain common symbiosis genes have been retained in different plant lineages that engage in intracellular symbiotic associations (Radhakrishnan et al. 2020). However, several transcriptome analyses in legumes show that a larger set of genes is induced in mycorrhized and nodulated roots (Manthey et al. 2004; Deguchi et al. 2007; Handa et al. 2015; Nanjareddy et al. 2017). For instance, mycorrhization and nodulation are affected in *Lotus* roots silenced or disrupted in the *Lectin Nucleotide Phosphohydrolases* (*LNP*), and the *SNARE* genes *LjVAMP72a* and *LjVAMP72b* (Roberts et al. 2013; Sogawa et al. 2018). We found that although the different fungal structures were formed in the *dahps1*-2 roots at 4 wpi, their numbers were significantly lower than those in Gifu. However, the proportion of the AM components in the root system was comparable to that of Gifu at 6 wpi, reflecting a delay in the colonization process. Such delay in the *dahps1*-2 mutant is probably linked to a deficiency of strigolactone levels, since these signaling molecules in the AM symbiosis are synthesized from carotenoids, compounds produced through the shikimate pathway. Additionally, the defects observed in the organization of the actin cytoskeleton of the cortical cells in the *dahps1* mutant could negatively impact the accommodation of arbuscules.

## Conclusion and perspectives

AAAs are the building blocks of indispensable metabolites and proteins for cell functioning. Interestingly, no pleiotropic effects were observed in the *dahps1*-1 and *dahps1*-2 mutants, but instead specific phenotypes in root hair development and mutualistic associations. The other 2 *DAHPS* expressed in *Lotus* roots could supply the minimal requirements of the cell but were still insufficient for the aforementioned processes. The linking of some AAAs and derived compounds with the cell wall and actin cytoskeleton is consistent with developmental and symbiotic defects observed in the *DAHPS1* mutant alleles. However, further research is needed to fully understand the specific metabolites and proteins involved in these different processes.

## Materials and methods

### Germination, nodulation kinetics, and genotyping

The genotypes used in this study belong to the genetic background of *L. japonicus* accession Gifu (Handberg and Stougaard 1992). For germination, the seedcoat was mechanically removed with sandpaper and the surface sterilized with sodium hypochlorite (3%, *w*/*v*) for 10 to 15 min and washed 3 to 5 times with sterile distilled water to remove traces of chlorine. The imbibed seeds were transferred to square Petri dishes with damp paper and incubated at 21 °C for germination. For nodulation assays, 3-to-5-d-old seedlings were transferred to square Petri dishes with a 1.4% (*w*/*v*) agar slant supplemented with ¼ B&D solution (Broughton and Dilworth 1971), which provides minimal requirements for plant growth without a nitrogen source to favor the RNS. To test the plant growth in nitrogen-replete conditions, the medium with agar was supplemented with ½ Gamborg’s B-5 basal medium (Sigma-Aldrich, G5893). The agar was covered with autoclaved filter paper and inoculated with *M. loti* R7A or with *A. pusense* IRBG74 (1 mL of bacterial culture per plate: OD600 = 0.05). The experiment was conducted in a temperature-controlled growth room (21 °C) with photoperiod (16/8 h). Using a stereomicroscope, the number of white and pink nodules was recorded weekly at 1 to 6 wk post inoculation (wpi). The LORE1 lines 30100225 and 30141487, affected in the *LjDAHPS1* gene, were obtained from the LORE1 mutant collection (Urbanski et al. 2012; Małolepszy et al. 2016) and genotyped to obtain homozygous mutants with allele-specific primers, following the database guidelines (Mun et al. 2016).

### Root phenotyping and pharmacological complementation

Gifu, *dahps1*-1, and *dahps1*-2 seeds were surface sterilized as mentioned above and transferred to square Petri dishes with an 0.8% (*w*/*v*) agar slant supplemented with 0.2× MS medium and 1% (*w*/*v*) sucrose, which allows optimal growth and contains a nitrogen source. For the chemical complementation, 50 and 100 *µ*M of L-phenylalanine, L-tryptophan, and L-tyrosine were added to the agar individually or in a mixture (50 and 100 *µ*M each AAA). The experiment was conducted in a growth chamber with controlled temperature and photoperiod. The root growth dynamics was analyzed by measuring the root length daily in the different genotypes, from the radicle emergence of up to 11 dpg. The lengths of the apical meristem and root hairs were measured from images obtained with a stereomicroscope on 10-dpg plants.

The actin cytoskeleton of 4-dpg seedlings of different genotypes was visualized with epifluorescence microscopy on root segments fixed with Alexa-Phalloidin (Yokota et al. 2009). The dynamics of filamentous actin plus ends was monitored in live root hairs of Gifu, *dahps1*-1, and *dahps1*-2 seedlings, mounted carefully in adapted Petri dishes with 1 mL of Fahraeus medium containing 4 *µ*L of Cyt-Fl probe (Molecular Probes; 2.5 *µ*M) (Zepeda et al. 2014). Cells incubated with Cyt-Fl were excited at 484 nm, and emission was collected at 530 nm (20-nm band pass). Similarly, live root hairs were incubated for 20 min with PI (20 to 40 *µ*M) to evaluate the distribution pattern of demethoxylated pectin, following the protocol described by Rounds et al. (2011). All filters used were from Chroma Technology, and image acquisition and analysis were carried out using MetaMorph/MetaFluor software (Universal Imaging, Molecular Devices).

### In silico analyses and RNAseq

The nucleotide and peptide sequences of LjDAHPS1, LjADAHPS2, and LjDAHPS3 were extracted from the *Lotus* genome browser (Mun *et al.* 2016; https://lotus.au.dk/genome/). The presence of the signal peptide in the amino acid sequences was predicted with TargetP—2.0 (https://services.healthtech.dtu.dk/service.php?TargetP-2.0). DAHPS from other plant species were obtained through protein BLAST in Phytozome (Goodstein et al. 2012), using as a query the 3 *Lotus* DAHPS. The amino acid sequences were aligned and bootstrapped (NJ tree, 1,000 iterations) by ClustalX 2, and the tree was visualized with Dendroscope (Huson and Scornavacca 2012). The accession numbers and annotations of DAHPS sequences are indicated in Supplemental Table S2.

The *dahps1*-2 seeds were germinated, and the seedlings were inoculated on plates with IRBG74 or mock treated (with water), following the protocol mentioned above. The root segments susceptible to intercellular infection (elongation and maturation zone) were cut and frozen in liquid nitrogen at 5 dpi for total RNA isolation. The RNA integrity and concentration were determined by gel electrophoresis and NanoDrop, respectively. DNA contamination was removed through DNAse treatment. Library preparations using randomly fragmented mRNA were performed by IMGM laboratories (Martinsried, Germany) and sequenced in paired-end 150-bp mode on an Illumina NovaSeq 6000 instrument. A decoy-aware index was constructed for Gifu transcripts using default Salmon parameters, and reads were quantified using the validate Mappings flag (Salmon version 0.14.1 (Patro et al. 2017)). Normalized expression levels and differential expression testing were calculated with the R-package DESeq2 version 1.20 (Love et al. 2014) after summarizing gene level abundance using the R-package tximport (version 1.8.0).

### Root infection phenotyping and nodule histology

The different genotypes were inoculated with the *M. loti*-LacZ strain, and the roots were harvested at 1 wpi for histochemical staining with X-Gal and recording the IT progression within the root tissues. The intercellular infection was analyzed as previously described (Montiel et al. 2021). Gifu and *dahps1*-2 roots were collected at 3 wpi with IRBG74, incubated for 1 min in a solution for surface disinfection (0.3% *w*/*v* of sodium chloride and 70% *v*/*v* EtOH), and then washed 5 times with distilled water. The total DNA was extracted from individual roots, adjusted to 10 ng *µ*L^−1^, and used as template for qPCR to evaluate the IRBG74 *NodA* abundance with the primers; forward: GAACTGCAAGTTGACGATCACGC and reverse: AAACGTCGTAACAAGCCCATGTGG. The expression values were normalized to the abundance of the *L. japonicus* gene LotjaGi1g1v0152000.1 with the oligonucleotides; forward: GAAGGACCCAGAGGATCACA and reverse: CGGTCTTCGTACTTCTTCGC using the delta Ct method (Pfaffl 2001).

Three-wk-old nodules were detached from Gifu and *dahps1*-2 plants inoculated with *M. loti* or IRBG74 and preserved in a fixative solution (0.1 M sodium cacodylate pH 7, 2.5% *v*/*v* glutaraldehyde). Fixed nodule slices were embedded in acrylic resin and sectioned for light microscopy and for TEM. For light microscopy analysis, the nodule semithin sections (1 *µ*m thickness) were stained with toluidine blue, while for TEM, the ultrathin sections (80 nm thickness) were stained with uranyl acetate (James and Sprent 1999; Madsen et al. 2010).

### Constructs for promoter activity and subcellular localization

The predicted promoter (2 kb upstream from the start codon) and CDS (1,617 bp) of *LjDAHPS1* were obtained from the *L. japonicus* Gifu genome (Kamal et al. 2020) in the *Lotus* database (Mun et al. 2016) and synthetized with 5´overghangs for goldengate cloning with *Bsa*I and *Bpi*I restriction sites replaced by silent mutations in the CDS. Promoter, CDS, fluorescent reporters, and terminator modules (Supplemental Table S5) were assembled and cloned into a pIV10 vector (Stougaard 1995), suitable for goldengate technology. *Agrobacterium rhizogenes* strain AR1193 was transformed with the *pDAHPS1::tYFP-nls* and *pDAHPS1::DAHPS1-YFP* constructs used to induce transgenic hairy roots in Gifu and *dahps1*-2 at 6 dpg, respectively, following a standardized protocol (Hansen et al. 1989). Three wk after infection, the main root was removed and the plants with hairy roots were transferred to square Petri dishes with ¼ B&D agar or plastic magenta boxes containing LECA substrate. Depending on the construct, the transgenic roots were mock treated or inoculated with either *M. loti*–DsRed (Kelly et al. 2013) or IRBG74–DsRed (Montiel et al. 2021) for inspection by confocal microscopy.

### Confocal microscopy of fluorescent reporters and symbiotic colonization

The confocal microscopy analysis was carried out in a Zeiss LSM780 microscope with excitation laser/emission filter (nm) settings adjusted to the fluorescent markers: autofluorescence, 405/408 to 498 nm; YFP, 514/517 to 560 nm; GFP, 514/517 to 540; and DsRed, 561/517 to 635 nm. Gifu and *dahps1*-2 plants were inoculated onto plates with the fluorescent-labeled *M. loti*–DsRed and IRBG74–DsRed strains. For AM analysis, the tissue was incubated for 4 h in EtOH (70% *v*/*v*) at room temperature, then transferred to KOH (20% *w*/*v*) for 2 to 3 d, and washed 3 times with water. Later, the root segments were treated with HCl (0.1 M) for 1 to 2 h and the solution was replaced by PBS containing 1 *µ*g mL^−1^ of WGA-Alexa Fluor 488 (Torabi et al. 2021). The root segments inoculated with the fluorescent bacteria, the transgenic roots harboring the *pDAHPS1::tYFP-nls* and *pDAHPS1::DAHPS1-YFP* constructs, and the tissue stained with WGA-Alexa Fluor 488 were mounted onto microscope slides for observation.

The analysis of root hairs after pharmacological treatments was performed on root segments from plants germinated and grown in media supplemented with AAAs, as mentioned above. The tissue was cleared using the ClearSee-adapted protocol (Kurihara et al. 2015). For cell wall visualization, the last step of the protocol consisted of 40-min incubation in ClearSee solution supplemented with 0.1% (*w*/*v*) of Calcofluor White, for which Fluorescent Brightener 28 disodium salt (Sigma-Aldrich F3397) was used. The roots were analyzed under a confocal laser scanning microscopy setup built around a Zeiss Axiovert 200M microscope (Oberkochen, Germany) that consisted of a high-speed galvo-resonant scanner for visible wavelengths (SCANVIS), a 405-nm laser OBIS 405nm LX 100mW laser system, 495-nm longpass dichroic, 440/40-nm bandpass emission filter, photomultiplier tube (PMT) modules (standard sensitivity), a Z-Axis piezo stage with controllers, and ThorImageLS 4.0 software. All parts were from Thorlabs Inc. (Newton, NJ, USA).

### Arbuscular mycorrhization quantification

Four-d-old seedlings of Gifu and *dahps1*-2 were placed between 2 discs of cellulose membrane filters (0.22 *µ*m pore size) with 50 to 100 spores of *R. intraradices* (Symbiom), previously resuspended in Long Ashton solution (Hewitt and Smith 1975) and the carrier substrate provided by the manufacturer. The filters with the inoculated plants were covered with autoclaved sand within Magenta boxes. At 4 and 6 wpi, the root system was detached from the plants and cleared as follows: 2% KOH *w*/*v* (1 h at 90 °C), 3 to 5 washes with distilled water, 2% HCl *v*/*v* (30 to 60 min), staining with trypan blue solution (1:1:1, lactic acid, glycerol, and water; 15 to 60 min at 90 °C) and washed with 50% (*v*/*v*) glycerol. Stained root segments were mounted onto microscope slides, and different fungal structures were recorded with the help of an optical microscope, following the protocol described by Trouvelot et al. (1986).

### Heterologous expression of *LjDAHPS1* in the *E. coli* NT1402 strain

The complementation plasmid pFAJ1708::*LjDAHPS1* was constructed by cloning the coding sequence of the *LjDAHPS1* transcriptionally fused to the constitutively active *nptII* promoter. Briefly, oligonucleotides LjDAHPS1_pFAJF (ATCTGATCAAGAGACAGGATATGGCTATCTCTTCCACTGCCA) and LjDAHPS1_pFAJR (ACGCGGGCCGCGGCGCGCCGGATCCTCACAGTCCTAAAGGGGCAAGAG) were used to PCR amplify *LjDAHPS1* from plasmid pL0M-SC3-DAHPS1, generating a DNA fragment with 20-bp overhangs at each end that facilitated Gibson assembly with *Xba*l/*BamH*I-digested pFAJ1708. Plasmid pFAJ1708::*DAHPS1* was transformed into chemically competent *E. coli* ST18 and selected on LB agar containing 50 *μ*g mL^−1^ 5-aminolevulinic acid and 15 *μ*g mL^−1^ tetracycline. pFAJ1708::DAHPS1 was then isolated from ST18, confirmed by sequencing and transformed directly into NT1402 by electroporation. Two clones were isolated, *E. coli* NT1402-LjDAHPS1_1 and *E. coli* NT1402-LjDAHPS1_2, which were utilized in all subsequent growth complementation assays.

### Statistical analyses

For multiple comparisons, analysis of variance (ANOVA) followed by Tukey’s post-hoc test was conducted. Individual comparisons were done by *t-*test or Mann–Whitney *U* test. *P*-values and the number of samples are shown in the figure legends.

### Accession numbers

IDs, sequences, and accession numbers of the genes analyzed in this study are shown in Supplemental Table S2. The RNAseq reads associated with this study are available in the SRA under bioproject accession number PRJNA632725.

## Supplementary Material

kiad398_Supplementary_Data

## Data Availability

The calculated expression values and statistics of the RNAseq data are included as Supplemental Tables S3 and S4.

## Supplemental data

The following materials are available in the online version of this article.

**Supplemental Figure S1.** Expression profile of *Lotus DAHPS* in different tissues and conditions.

**Supplemental Figure S2.** Multiple sequence alignment of *Lotus* DAHPS.

**Supplemental Figure S3.** Root growth phenotype and shoot length of *dahps1*-1 and *dahps1*-2 mutants.

**Supplemental Figure S4.** Actin cytoskeleton organization in epidermal and cortical cells of *dahps1* mutants.

**Supplemental Figure S5.** Partial heterologous complementation of an *E. coli dahps* triple mutant by *LjDAHPS1*.

**Supplemental Figure S6.** Chemical complementation of *dahps1*-2 mutant and subcellular localization of DAHPS1-YFP in *Lotus* transgenic roots.

**Supplemental Figure S7.** DEG in uninoculated 5-d-old roots of the *dahps1*-2 mutant.

**Supplemental Figure S8.** Nodule formation and plant growth in the *dahps1*-1 and *dahps1*-2 mutants.

**Supplemental Table S1.** List of DEGs in *Lotus* roots after *M. loti* or IRBG7 inoculation.

**Supplemental Table S2.** Peptide sequences of DAHPS in various plant species.

**Supplemental Table S3.** DEGs in uninoculated *dahps1*-2 roots.

**Supplemental Table S4.** DEGs at 5 dpi with IRBG74 in *dahps1*-2 roots.

**Supplemental Table S5.** Sequences used for goldengate cloning.

## References

[kiad398-B1] Baumberger N , Steiner M, Ryser U, Keller B, Ringli C. Synergistic interaction of the two paralogous Arabidopsis genes LRX1 and LRX2 in cell wall formation during root hair development. Plant J. 2003:35(1):71–81. 10.1046/j.1365-313X.2003.01784.x 12834403

[kiad398-B2] Bek AS , Sauer J, Thygesen MB, Duus JØ, Petersen BO, Thirup S, James E, Jensen KJ, Stougaard J, Radutoiu S. Improved characterization of nod factors and genetically based variation in LysM Receptor domains identify amino acids expendable for nod factor recognition in Lotus spp. Mol Plant Microbe Interact. 2010:23(1):58–66. 10.1094/MPMI-23-1-0058 19958139

[kiad398-B3] Bonfante P , Genre A. Mechanisms underlying beneficial plant-fungus interactions in mycorrhizal symbiosis. Nat Commun. 2010:1(1):48. 10.1038/ncomms1046 20975705

[kiad398-B4] Broughton WJ , Dilworth MJ. Control of leghaemoglobin synthesis in snake beans. Biochemical J. 1971:125(4):1075–1080. 10.1042/bj1251075 PMC1178271 5144223

[kiad398-B5] Cárdenas L , Thomas-Oates JE, Nava N, López-Lara IM, Hepler PK, Quinto C. The role of nod factor substituents in actin cytoskeleton rearrangements in *Phaseolus vulgaris*. Mol Plant Microbe Interact. 2003:16(4):326–334. 10.1094/MPMI.2003.16.4.326 12744461

[kiad398-B6] Copeland C . Same but different: examining the molecular mechanisms of intercellular rhizobial infection. Plant Physiol. 2021:185(3):754–756. 10.1093/plphys/kiaa097 33822221 PMC8133676

[kiad398-B7] Cummings SP , Gyaneshwar P, Vinuesa P, Farruggia FT, Andrews M, Humphry D, Elliott GN, Nelson A, Orr C, Pettitt D, et al Nodulation of Sesbania species by Rhizobium (Agrobacterium) strain IRBG74 and other rhizobia. Environ Microbiol. 2009:11(10):2510–2525. 10.1111/j.1462-2920.2009.01975.x 19555380 PMC7163632

[kiad398-B8] Deguchi Y , Banba M, Shimoda Y, Chechetka SA, Suzuri R, Okusako Y, Ooki Y, Toyokura K, Suzuki A, Uchiumi T, et al Transcriptome profiling of *Lotus japonicus* roots during arbuscular mycorrhiza development and comparison with that of nodulation. DNA Res. 2007:14(3):117–133. 10.1093/dnares/dsm014 17634281 PMC2779901

[kiad398-B9] Downie JA . Legume nodulation. Curr Biol. 2014:24(5):R184–R190. 10.1016/j.cub.2014.01.028 24602880

[kiad398-B10] Fonseca-García C , Nava N, Lara M, Quinto C. An NADPH oxidase regulates carbon metabolism and the cell cycle during root nodule symbiosis in common bean (*Phaseolus vulgaris*). BMC Plant Biol. 2021:21(1):274. 10.1186/s12870-021-03060-z 34130630 PMC8207584

[kiad398-B11] Gayomba SR , Muday GK. Flavonols regulate root hair development by modulating accumulation of reactive oxygen species in the root epidermis. Development. 2020:147(8):dev185819. 10.1242/dev.185819 32179566

[kiad398-B12] Geng P , Zhang S, Liu J, Zhao C, Wu J, Cao Y, Fu C, Han X, He H, Zhao Q. MYB20, MYB42, MYB43, and MYB85 regulate phenylalanine and lignin biosynthesis during secondary cell wall formation. Plant Physiol. 2020:182(3):1272–1283. 10.1104/pp.19.01070 31871072 PMC7054866

[kiad398-B13] Goodstein DM , Shu S, Howson R, Neupane R, Hayes RD, Fazo J, Mitros T, Dirks W, Hellsten U, Putnam N, et al Phytozome: a comparative platform for green plant genomics. Nucleic Acids Res. 2012:40(D1):D1178–D1186. 10.1093/nar/gkr944 22110026 PMC3245001

[kiad398-B14] Guillen G , Valdes-Lopez V, Noguez R, Olivares J, Rodriguez-Zapata LC, Perez H, Vidali L, Villanueva MA, Sanchez F. Profilin in *Phaseolus vulgaris* is encoded by two genes (only one expressed in root nodules) but multiple isoforms are generated in vivo by phosphorylation on tyrosine residues. Plant J. 1999:19(5):497–508. 10.1046/j.1365-313X.1999.00542.x 10504572

[kiad398-B15] Handa Y , Nishide H, Takeda N, Suzuki Y, Kawaguchi M, Saito K. RNA-seq transcriptional profiling of an arbuscular mycorrhiza provides insights into regulated and coordinated gene expression in *Lotus japonicus* and *Rhizophagus irregularis*. Plant Cell Physiol. 2015:56(8):1490–1511. 10.1093/pcp/pcv071 26009592

[kiad398-B16] Handberg K , Stougaard J. *Lotus japonicus*, an autogamous, diploid legume species for classical and molecular genetics. Plant J. 1992:2(4):487–496. 10.1111/j.1365-313X.1992.00487.x

[kiad398-B17] Hansen J , Jørgensen JE, Stougaard J, Marcker KA. Hairy roots—a short cut to transgenic root nodules. Plant Cell Rep. 1989:8(1):12–15. 10.1007/BF00735768 24232586

[kiad398-B18] Hewitt EJ , Smith TA. Plant mineral nutrition. London: English U.P.; 1975.

[kiad398-B19] Hossain MS , Liao J, James EK, Sato S, Tabata S, Jurkiewicz A, Madsen LH, Stougaard J, Ross L, Szczyglowski K. *Lotus japonicus* ARPC1 is required for rhizobial infection. Plant Physiol. 2012:160(2):917–928. 10.1104/pp.112.202572 22864583 PMC3461565

[kiad398-B20] Huson DH , Scornavacca C. Dendroscope 3: an interactive tool for rooted phylogenetic trees and networks. Syst Biol. 2012:61(6):1061–1067. 10.1093/sysbio/sys062 22780991

[kiad398-B21] James EK , Sprent JI. Development of N2-fixing nodules on the wetland legume *Lotus uliginosus* exposed to conditions of flooding. New Phytol. 1999:142(2):219–231. 10.1046/j.1469-8137.1999.00394.x

[kiad398-B22] Jayaraman K , Trachtmann N, Sprenger GA, Gohlke H. Protein engineering for feedback resistance in 3-deoxy-D-arabino-heptulosonate 7-phosphate synthase. Appl Microbiol Biotechnol. 2022:106(19–20):6505–6517. 10.1007/s00253-022-12166-9 36109385 PMC9529685

[kiad398-B23] Kamal N , Mun T, Reid D, Lin JS, Akyol TY, Sandal N, Asp T, Hirakawa H, Stougaard J, Mayer KFX, et al Insights into the evolution of symbiosis gene copy number and distribution from a chromosome-scale *Lotus japonicus* Gifu genome sequence. DNA Res. 2020:27(3):dsaa015. 10.1093/dnares/dsaa015 PMC7508351 32658273

[kiad398-B24] Kameyama K , Kishi Y, Yoshimura M, Kanzawa N, Sameshima M, Tsuchiya T. Tyrosine phosphorylation in plant bending. Nature. 2000:407(6800):37. 10.1038/35024149 10993062

[kiad398-B25] Karas B , Murray J, Gorzelak M, Smith A, Sato S, Tabata S, Szczyglowski K. Invasion of *Lotus japonicus* root hairless 1 by *Mesorhizobium loti* involves the nodulation factor-dependent induction of root hairs. Plant Physiol. 2005:137(4):1331–1344. 10.1104/pp.104.057513 15778455 PMC1088324

[kiad398-B26] Karas BJ , Ross L, Novero M, Amyot L, Shrestha A, Inada S, Nakano M, Sakai T, Bonetta D, Sato S, et al Intragenic complementation at the *Lotus japonicus* CELLULOSE SYNTHASE-LIKE D1 locus rescues root hair defects. Plant Physiol. 2021:186(4):2037–2050. 10.1093/plphys/kiab204 34618101 PMC8331140

[kiad398-B27] Ke D , Li X, Han Y, Cheng L, Yuan H, Wang L. ROP6 Is involved in root hair deformation induced by Nod factors in *Lotus japonicus*. Plant Physiol Biochem. 2016:108:488–498. 10.1016/j.plaphy.2016.08.015 27592173

[kiad398-B28] Kelly SJ , Muszyński A, Kawaharada Y, Hubber AM, Sullivan JT, Sandal N, Carlson RW, Stougaard J, Ronson CW. Conditional requirement for exopolysaccharide in the Mesorhizobium-Lotus symbiosis. Mol Plant Microbe Interact. 2013:26(3):319–329. 10.1094/MPMI-09-12-0227-R 23134480

[kiad398-B29] Kurihara D , Mizuta Y, Sato Y, Higashiyama T. Clearsee: a rapid optical clearing reagent for whole-plant fluorescence imaging. Development. 2015:142(23):4168–4179. 10.1242/dev.127613 26493404 PMC4712841

[kiad398-B30] Lei MJ , Wang Q, Li X, Chen A, Luo L, Xie Y, Li G, Luo D, Mysore KS, Wen J, et al The small GTPase ROP10 of *Medicago truncatula* is required for both tip growth of root hairs and nod factor-induced root hair deformation. Plant Cell. 2015:27(3):806–822. 10.1105/tpc.114.135210 25794934 PMC4558664

[kiad398-B31] Lemaître C , Bidet P, Benoist JF, Schlemmer D, Sobral E, d'Humières C, Bonacorsi S. The ssbL gene harbored by the ColV plasmid of an Escherichia coli neonatal meningitis strain is an auxiliary virulence factor boosting the production of siderophores through the shikimate pathway. J Bacteriol. 2014:196(7):1343–1349. 10.1128/JB.01153-13 24443535 PMC3993347

[kiad398-B32] Lévy J , Bres C, Geurts R, Chalhoub B, Kulikova O, Duc G, Journet EP, Ané JM, Lauber E, Bisseling T, et al A putative Ca2+ and calmodulin-dependent protein kinase required for bacterial and fungal symbioses. Science. 2004:303(5662):1361–1364. 10.1126/science.1093038 14963335

[kiad398-B33] Liu J , Liu MX, Qiu LP, Xie F. SPIKE1 activates the GTPase ROP6 to guide the polarized growth of infection threads in *Lotus japonicus*. Plant Cell. 2020:32(12):3774–3791. 10.1105/tpc.20.00109 33023954 PMC7721321

[kiad398-B34] Liu CW , Murray JD. The role of flavonoids in nodulation host-range specificity: an update. Plants (Basel). 2016:5(3):33. 10.3390/plants5030033 27529286 PMC5039741

[kiad398-B35] Love MI , Huber W, Anders S. Moderated estimation of fold change and dispersion for RNA-seq data with DESeq2. Genome Biol. 2014:15(12):550. 10.1186/s13059-014-0550-8 25516281 PMC4302049

[kiad398-B36] MacLean AM , Bravo A, Harrison MJ. Plant signaling and metabolic pathways enabling arbuscular mycorrhizal symbiosis. Plant Cell. 2017:29(10):2319–2335. 10.1105/tpc.17.00555 28855333 PMC5940448

[kiad398-B37] Madsen LH , Tirichine L, Jurkiewicz A, Sullivan JT, Heckmann AB, Bek AS, Ronson CW, James EK, Stougaard J. The molecular network governing nodule organogenesis and infection in the model legume *Lotus japonicus*. Nat Commun. 2010:1(1):10. 10.1038/ncomms1009 20975672 PMC2892300

[kiad398-B38] Małolepszy A , Mun T, Sandal N, Gupta V, Dubin M, Urbański D, Shah N, Bachmann A, Fukai E, Hirakawa H, et al The LORE1 insertion mutant resource. Plant J. 2016:88(2):306–317. 10.1111/tpj.13243 27322352

[kiad398-B39] Manthey K , Krajinski F, Hohnjec N, Firnhaber C, Pü A, Perlick AM, Küster H. Transcriptome profiling in root nodules and arbuscular mycorrhiza identifies a collection of novel genes induced during *Medicago truncatula* root endosymbioses. Mol Plant Microbe Interact. 2004:17(10):1063–1077. 10.1094/MPMI.2004.17.10.1063 15497399

[kiad398-B40] Mergaert P , Kereszt A, Kondorosi E. Gene expression in nitrogen-fixing symbiotic nodule cells in *Medicago truncatula* and other nodulating plants. Plant Cell. 2020:32(1):42–68. 10.1105/tpc.19.00494 31712407 PMC6961632

[kiad398-B41] Mitra S , Mukherjee A, Wiley-Kalil A, Das S, Owen H, Reddy PM, Ané JM, James EK, Gyaneshwar P. A rhamnose-deficient lipopolysaccharide mutant of Rhizobium sp. IRBG74 is defective in root colonization and beneficial interactions with its flooding-tolerant hosts *Sesbania cannabina* and wetland rice. J Exp Bot. 2016:67(19):5869–5884. 10.1093/jxb/erw354 27702995

[kiad398-B42] Montiel J , Reid D, Grønbaek TH, Benfeldt CM, James EK, Ott T, Ditengou FA, Nadzieja M, Kelly S, Stougaard J. Distinct signaling routes mediate intercellular and intracellular rhizobial infection in *Lotus japonicus*. Plant Physiol. 2021:185(3):1131–1147. 10.1093/plphys/kiaa049 33793909 PMC8133683

[kiad398-B43] Mun T , Bachmann A, Gupta V, Stougaard J, Andersen SU. Lotus Base: an integrated information portal for the model legume *Lotus japonicus*. Sci Rep. 2016:6(1):39447. 10.1038/srep39447 28008948 PMC5180183

[kiad398-B44] Murray JD , Muni RR, Torres-Jerez I, Tang Y, Allen S, Andriankaja M, Li G, Laxmi A, Cheng X, Wen J, et al Vapyrin, a gene essential for intracellular progression of arbuscular mycorrhizal symbiosis, is also essential for infection by rhizobia in the nodule symbiosis of *Medicago truncatula*. Plant J. 2011:65(2):244–252. 10.1111/j.1365-313X.2010.04415.x 21223389

[kiad398-B45] Nanjareddy K , Arthikala MK, Gómez BM, Blanco L, Lara M. Differentially expressed genes in mycorrhized and nodulated roots of common bean are associated with defense, cell wall architecture, N metabolism, and P metabolism. PLoS One. 2017:12(8):e0182328. 10.1371/journal.pone.0182328 PMC5542541 28771548

[kiad398-B46] Oldroyd GE . Speak, friend, and enter: signalling systems that promote beneficial symbiotic associations in plants. Nat Rev Microbiol. 2013:11(4):252–263. 10.1038/nrmicro2990 23493145

[kiad398-B47] Parniske M . Arbuscular mycorrhiza: the mother of plant root endosymbioses. Nat Rev Microbiol. 2008:6(10):763–775. 10.1038/nrmicro1987 18794914

[kiad398-B48] Patro R , Duggal G, Love MI, Irizarry RA, Kingsford C. Salmon provides fast and bias-aware quantification of transcript expression. Nat Methods. 2017:14(4):417–419. 10.1038/nmeth.4197 28263959 PMC5600148

[kiad398-B49] Pfaffl MW . A new mathematical model for relative quantification in real-time RT-PCR. Nucleic Acids Res. 2001:29(9):e45. 10.1093/nar/29.9.e45 11328886 PMC55695

[kiad398-B50] Qiu L , Lin JS, Xu J, Sato S, Parniske M, Wang TL, Downie JA, Xie F. SCARN A novel class of SCAR protein that is required for root-hair infection during legume nodulation. PLoS Genet. 2015:11(10):e1005623. 10.1371/journal.pgen.1005623 PMC4627827 26517270

[kiad398-B51] Quilbé J , Montiel J, Arrighi JF, Stougaard J. Molecular mechanisms of intercellular rhizobial infection: novel findings of an ancient process. Front Plant Sci. 2022:13:922982. 10.3389/fpls.2022.922982 PMC9260380 35812902

[kiad398-B52] Radhakrishnan GV , Keller J, Rich MK, Vernie T, Mbadinga Mbadinga DL, Vigneron N, Cottret L, Clemente HS, Libourel C, Cheema J, et al An ancestral signalling pathway is conserved in intracellular symbioses-forming plant lineages. Nat Plants. 2020:6(3):280–289. 10.1038/s41477-020-0613-7 32123350

[kiad398-B53] Ringli C , Baumberger N, Diet A, Frey B, Keller B. ACTIN2 is essential for bulge site selection and tip growth during root hair development of Arabidopsis. Plant Physiol. 2002:129(4):1464–1472. 10.1104/pp.005777 12177460 PMC166735

[kiad398-B54] Roberts NJ , Morieri G, Kalsi G, Rose A, Stiller J, Edwards A, Xie F, Gresshoff PM, Oldroyd GE, Downie JA, et al Rhizobial and mycorrhizal symbioses in *Lotus japonicus* require lectin nucleotide phosphohydrolase, which acts upstream of calcium signaling. Plant Physiol. 2013:161(1):556–567. 10.1104/pp.112.206110 23136382 PMC3532285

[kiad398-B55] Rounds CM , Lubeck E, Hepler PK, Winship LJ. Propidium iodide competes with Ca(2+) to label pectin in pollen tubes and Arabidopsis root hairs. Plant Physiol. 2011:157(1):175–187. 10.1104/pp.111.182196 21768649 PMC3165868

[kiad398-B56] Roy S , Liu W, Nandety RS, Crook A, Mysore KS, Pislariu CI, Frugoli J, Dickstein R, Udvardi MK. Celebrating 20 years of genetic discoveries in legume nodulation and symbiotic nitrogen fixation([OPEN]). Plant Cell. 2020:32(1):15–41. 10.1105/tpc.19.00279 31649123 PMC6961631

[kiad398-B57] Schauser L , Roussis A, Stiller J, Stougaard J. A plant regulator controlling development of symbiotic root nodules. Nature. 1999:402(6758):191–195. 10.1038/46058 10647012

[kiad398-B58] Schiessl K , Lilley JLS, Lee T, Tamvakis I, Kohlen W, Bailey PC, Thomas A, Luptak J, Ramakrishnan K, Carpenter MD, et al NODULE INCEPTION recruits the lateral root developmental program for symbiotic nodule organogenesis in Medicago truncatula. Curr Biol. 2019:29(21):3657–3668 e5. 10.1016/j.cub.2019.09.005 31543454 PMC6839406

[kiad398-B59] Seifert GJ , Barber C, Wells B, Dolan L, Roberts K. Galactose biosynthesis in Arabidopsis: genetic evidence for substrate channeling from UDP-D-galactose into cell wall polymers. Curr Biol. 2002:12(21):1840–1845. 10.1016/S0960-9822(02)01260-5 12419184

[kiad398-B60] Shibata M , Sugimoto K. A gene regulatory network for root hair development. J Plant Res. 2019:132(3):301–309. 10.1007/s10265-019-01100-2 30903397 PMC7082380

[kiad398-B61] Shimamura M , Kumaki T, Hashimoto S, Saeki K, Ayabe SI, Higashitani A, Akashi T, Sato S, Aoki T. Phenolic acids induce nod factor production in *Lotus japonicus*-Mesorhizobium symbiosis. Microbes Environ. 2022:37(1):ME21094. 10.1264/jsme2.ME21094 PMC8958295 35283370

[kiad398-B62] Simpson JP , Olson J, Dilkes B, Chapple C. Identification of the tyrosine- and phenylalanine-derived soluble metabolomes of Sorghum. Front Plant Sci. 2021:12:714164. 10.3389/fpls.2021.714164 PMC8476951 34594350

[kiad398-B63] Sinharoy S , Liu C, Breakspear A, Guan D, Shailes S, Nakashima J, Zhang S, Wen J, Torres-Jerez I, Oldroyd G, et al A Medicago truncatula cystathionine-beta-synthase-like domain-containing protein is required for rhizobial infection and symbiotic nitrogen fixation. Plant Physiol. 2016:170(4):2204–2217. 10.1104/pp.15.01853 26884486 PMC4825145

[kiad398-B64] Sogawa A , Yamazaki A, Yamasaki H, Komi M, Manabe T, Tajima S, Hayashi M, Nomura M. SNARE proteins LjVAMP72a and LjVAMP72b are required for root symbiosis and root hair formation in *Lotus japonicus*. Front Plant Sci. 2018:9:1992. 10.3389/fpls.2018.01992 30700990 PMC6343493

[kiad398-B65] Sprent JI . Evolving ideas of legume evolution and diversity: a taxonomic perspective on the occurrence of nodulation. New Phytol. 2007:174(1):11–25. 10.1111/j.1469-8137.2007.02015.x 17335493

[kiad398-B66] Stougaard J . *Agobacterium rhizogenes* as a vector for transforming higher plants. Methods Mol Biol. 1995:49:49–61. 10.1385/0-89603-321-X:49 8563829

[kiad398-B67] Stracke S , Kistner C, Yoshida S, Mulder L, Sato S, Kaneko T, Tabata S, Sandal N, Stougaard J, Szczyglowski K, et al A plant receptor-like kinase required for both bacterial and fungal symbiosis. Nature. 2002:417(6892):959–962. 10.1038/nature00841 12087405

[kiad398-B68] Su C , Zhang G, Rodriguez-Franco M, Hinnenberg R, Wietschorke J, Liang P, Yang W, Uhler L, Li X, Ott T. Transcellular progression of infection threads in *Medicago truncatula* roots is associated with locally confined cell wall modifications. Curr Biol. 2023:33(3):533–542.e5. 10.1016/j.cub.2022.12.051 36657449 PMC9937034

[kiad398-B69] Subramanian S , Stacey G, Yu O. Endogenous isoflavones are essential for the establishment of symbiosis between soybean and *Bradyrhizobium japonicum*. Plant J. 2006:48(2):261–273. 10.1111/j.1365-313X.2006.02874.x 17018035

[kiad398-B70] Torabi S , Varshney K, Villaecija-Aguilar JA, Keymer A, Gutjahr C. Controlled assays for phenotyping the effects of strigolactone-like molecules on arbuscular mycorrhiza development. Methods Mol Biol. 2021:2309:157–177. 10.1007/978-1-0716-1429-7_13 34028686

[kiad398-B71] Trouvelot A , Kough JL, Gianinazzi-Pearson V. Mesure du taux de mycorhization VA d’un système radiculaire. Recherche de méthodes d’estimation ayant une signification fonctionnelle. Proceedings of the 1st European Symposium on Mycorrhizae. 1986:217–221.

[kiad398-B72] Tzin V , Galili G. New insights into the shikimate and aromatic amino acids biosynthesis pathways in plants. Mol Plant. 2010:3(6):956–972. 10.1093/mp/ssq048 20817774

[kiad398-B73] Udvardi M , Poole PS. Transport and metabolism in legume-rhizobia symbioses. Annu Rev Plant Biol. 2013:64(1):781–805. 10.1146/annurev-arplant-050312-120235 23451778

[kiad398-B74] Urbanski DF , Malolepszy A, Stougaard J, Andersen SU. Genome-wide LORE1 retrotransposon mutagenesis and high-throughput insertion detection in *Lotus japonicus*. Plant J. 2012:69(4):731–741. 10.1111/j.1365-313X.2011.04827.x 22014280

[kiad398-B75] Vogt T . Phenylpropanoid biosynthesis. Mol Plant. 2010:3(1):2–20. 10.1093/mp/ssp106 20035037

[kiad398-B76] Wang X , Cnops G, Vanderhaeghen R, De Block S, Van Montagu M, Van Lijsebettens M. AtCSLD3, a cellulose synthase-like gene important for root hair growth in Arabidopsis. Plant Physiol. 2001:126(2):575–586. 10.1104/pp.126.2.575 11402188 PMC111150

[kiad398-B77] Wasson AP , Pellerone FI, Mathesius U. Silencing the flavonoid pathway in *Medicago truncatula* inhibits root nodule formation and prevents auxin transport regulation by rhizobia. Plant Cell. 2006:18(7):1617–1629. 10.1105/tpc.105.038232 16751348 PMC1488924

[kiad398-B78] Yano K , Yoshida S, Muller J, Singh S, Banba M, Vickers K, Markmann K, White C, Schuller B, Sato S, et al CYCLOPS, a mediator of symbiotic intracellular accommodation. Proc Natl Acad Sci U S A. 2008:105(51):20540–20545. 10.1073/pnas.0806858105 19074278 PMC2629324

[kiad398-B79] Yokota K , Fukai E, Madsen LH, Jurkiewicz A, Rueda P, Radutoiu S, Held M, Hossain MS, Szczyglowski K, Morieri G, et al Rearrangement of actin cytoskeleton mediates invasion of *Lotus japonicus* roots by *Mesorhizobium loti*. Plant Cell. 2009:21(1):267–284. 10.1105/tpc.108.063693 19136645 PMC2648097

[kiad398-B80] Zepeda I , Sanchez-Lopez R, Kunkel JG, Banuelos LA, Hernandez-Barrera A, Sanchez F, Quinto C, Cardenas L. Visualization of highly dynamic F-actin plus ends in growing *phaseolus vulgaris* root hair cells and their responses to *Rhizobium etli* nod factors. Plant Cell Physiol. 2014:55(3):580–592. 10.1093/pcp/pct202 24399235

[kiad398-B81] Zi H , Xiang Y, Li M, Wang T, Ren H. Reversible protein tyrosine phosphorylation affects pollen germination and pollen tube growth via the actin cytoskeleton. Protoplasma. 2007:230(3–4):183–191. 10.1007/s00709-006-0232-9 17458633

