# Supplementary material for: Aromatic amino acid biosynthesis impacts root hair development and symbiotic associations in Lotus japonicus
Source: Plant Physiol. 2023 Jul 10;193(2):1508–26. doi: 10.1093/plphys/kiad398 (PMC10517252; doi:10.1093/plphys/kiad398)
Supplement: kiad398_Supplementary_Data [file kiad398_supplementary_data.zip › Supplemental Figures.pdf]

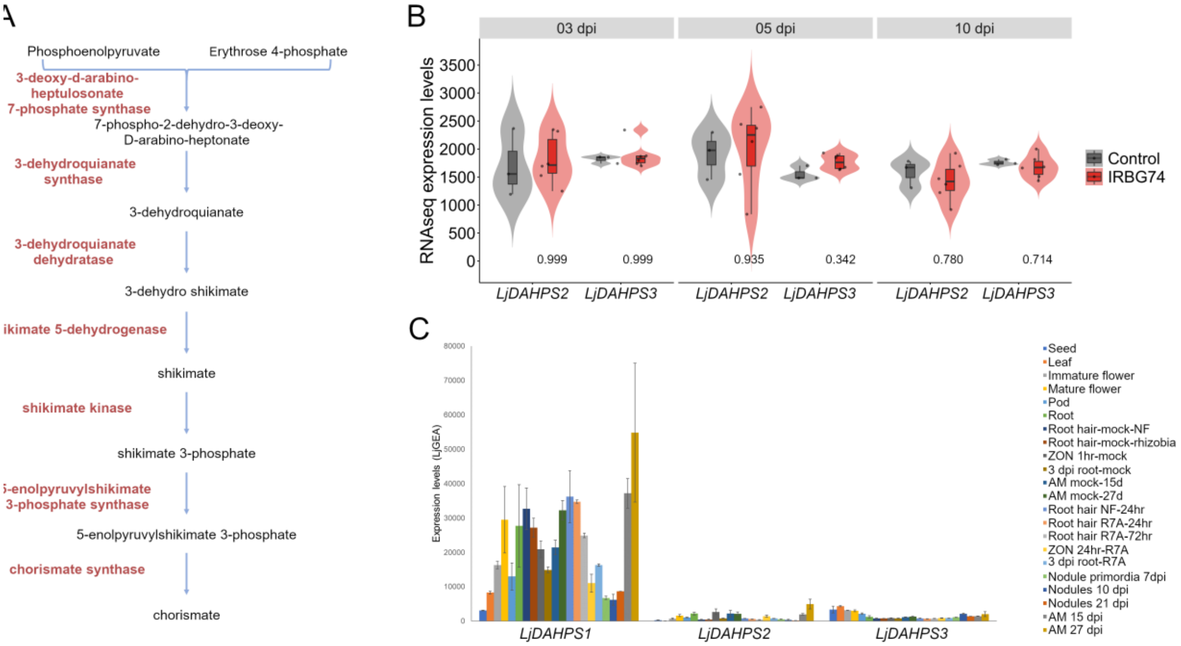

Supplemental Figure S1. Expression profile of *Lotus DAHPS* in different tissues and conditions. (**A**) Compounds (black) and enzymes (red) involved in the shikimate pathway. (**B**) Expression levels of *LjDAHPS2* and *LjDAHPS3* extracted from RNAseq data of *Lotus* roots at 3, 5 and 10 dpi with IRBG74 or mock-treated (control). Violin boxplots: center line, median; box limits, upper and lower quartiles; whiskers, 1.5× interquartile range; points, individual data points. *p*-adjusted values obtained from the RNAseq data are shown below the violin boxplots. (**C**) Expression levels of *LjDAHPS1*, *LjDAHPS2* and *LjDAHPS3* in different tissues, organs and in response to different stimuli. Data extracted from the *Lotus japonicus* Expression Atlas (https://lotus.au.dk/expat/). Error bars indicate SD. Further details for each condition can be found in the *Lotus japonicus* Expression Atlas.

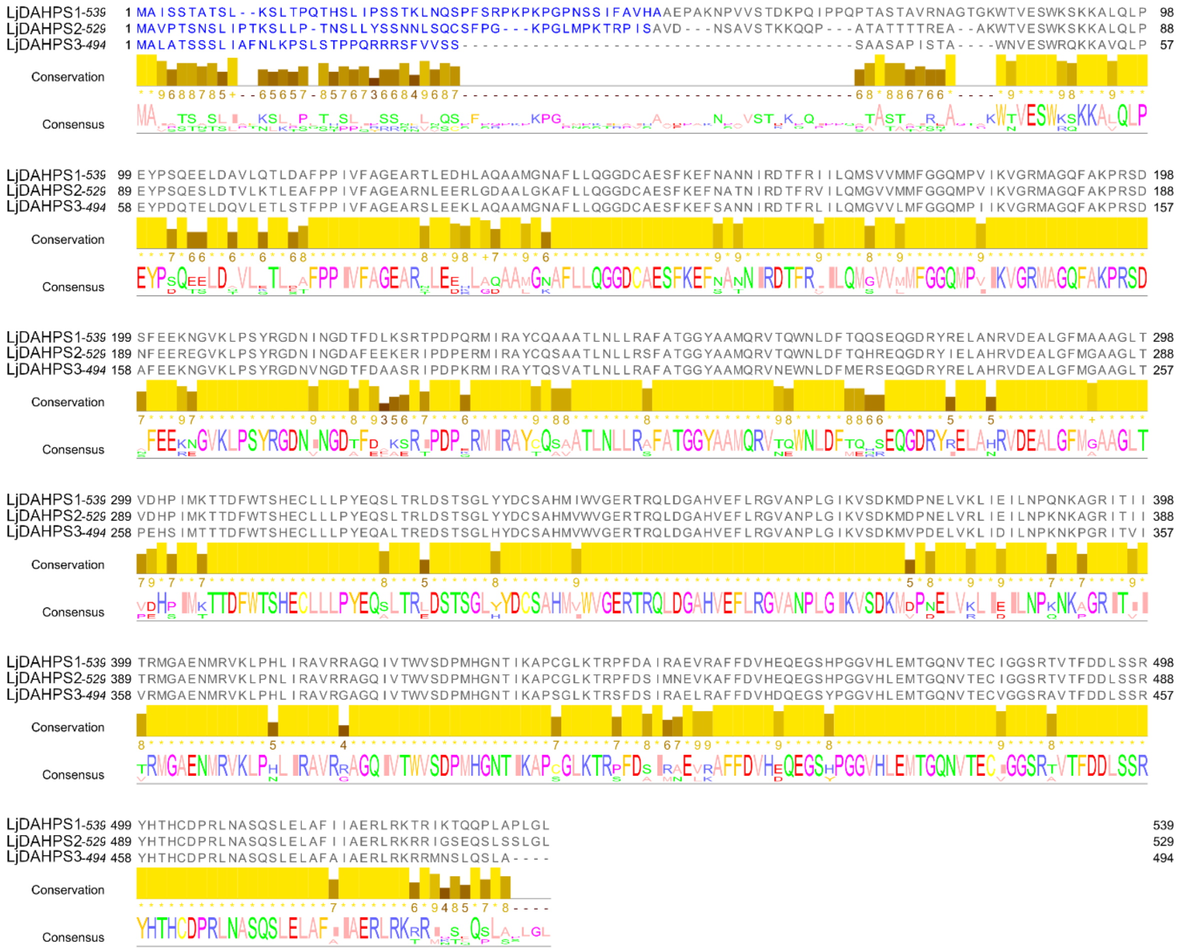

Supplemental Figure S2. Multiple sequence alignment of *Lotus* DAHPS. Multiple alignment of deduced full peptide sequences of LjDAHPS1, LjDAHPS2 and LjDAHPS3. In blue, residues of the predicted plastid signal peptide.

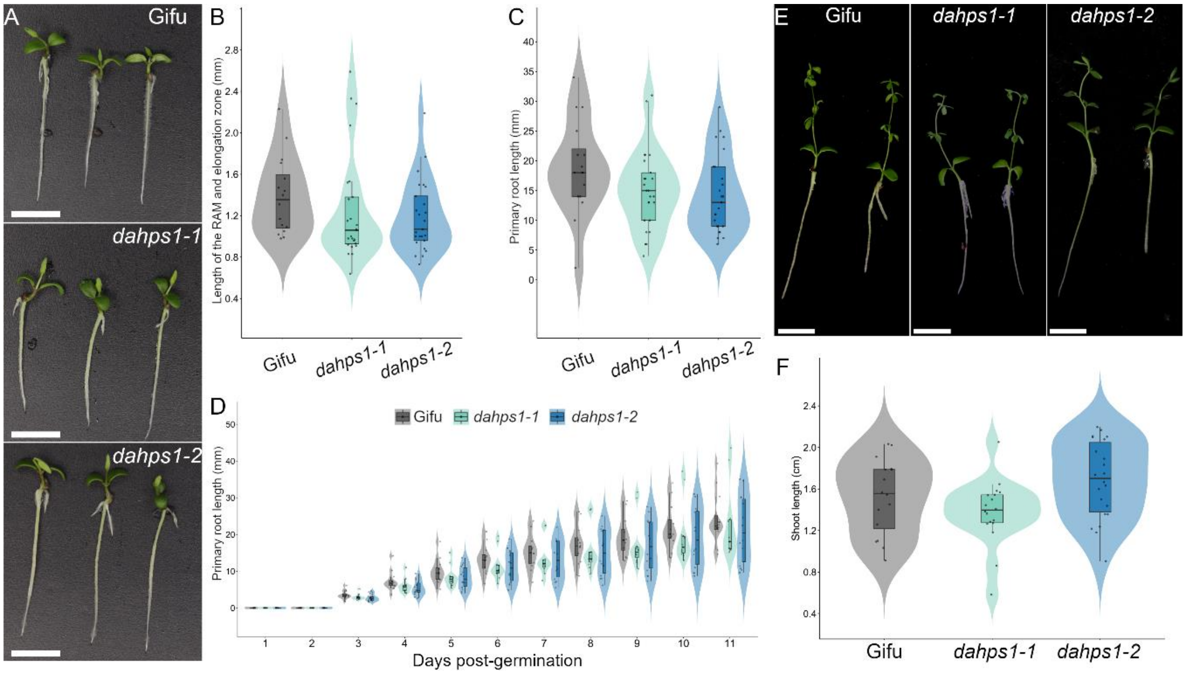

Supplemental Figure S3. Root growth phenotype and shoot length of *dahps1*-1 and *dahps1*-2 mutants. Representative images (**A**), length of the root apical meristem (RAM) with the elongation zone (**B**), and length of the primary root (**C**) in 10 dpg Gifu, *dahps1*-1 and *dahps1*-2 plants, grown in MS medium. (**D**) Root growth dynamics of Gifu, *dahps1*-1 and *dahps1*-2 at 1-10 dpg. Gifu w. t. (n= 12) and the *dahps1*-1 (n= 11), and *dahps1*-2 (n= 14) mutants. Representative images (**E**) and shoot length (**F**) at 5 wpg of Gifu and *dahps1* plants grown in B5 medium containing 12 mM of KNO3. Violin boxplots: center line, median; box limits, upper and lower quartiles; whiskers, 1.5× interquartile range; points, individual data points. No significant differences were obtained for the different parameters when compared to Gifu by ANOVA followed by Tukey's HSD test. Scale, 1 cm.

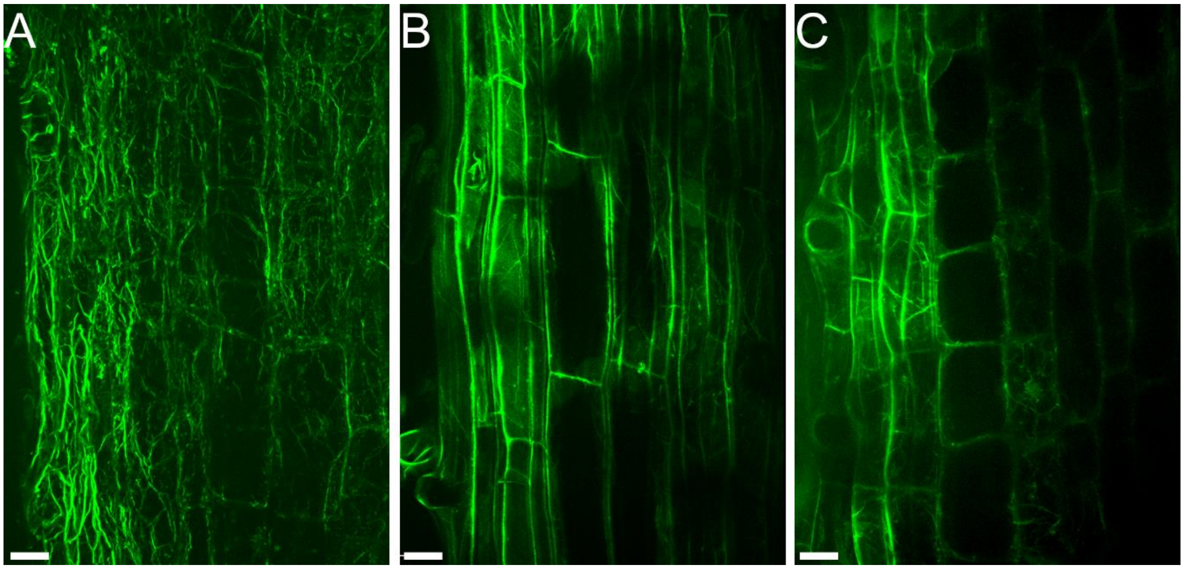

Supplemental Figure S4. Actin cytoskeleton organization in epidermal and cortical cells of *dahps1* mutants. Visualization of actin microfilaments organization by Alexa-Phalloidin staining in the epidermal and cortical root cells of Gifu (A) and *dahps1* plants (B and C) at 5 dpg. Scale, 20 µm.

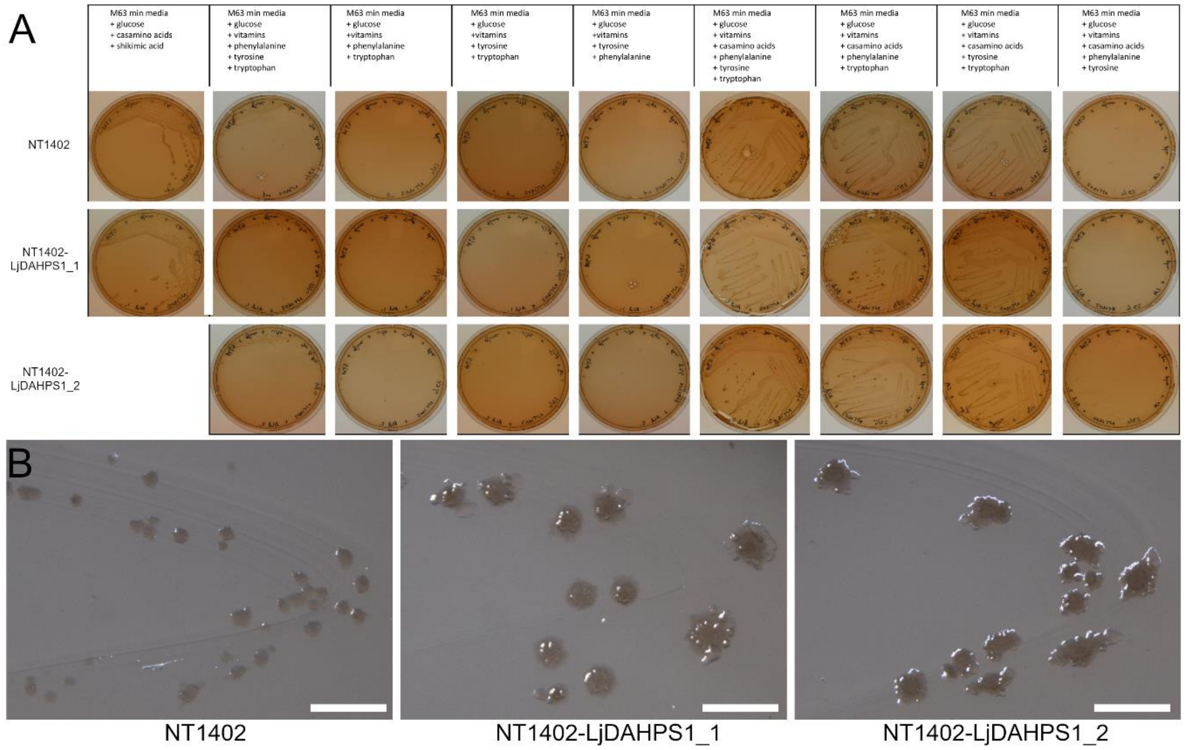

Supplemental Figure S5. Partial heterologous complementation of an *E. coli dahps* triple mutant by the *LjDAHPS1*. (A) Representative images of colonies formed on Petri dishes by the triple mutant in *DAHPS* genes NT1402 and two derivative strains harbouring the LjDAHPS1 sequence (NT1402-LjDAHPS1_1 and NT1402-LjDAHPS1_2). The *E. coli* strains were grown 18 days on M63 minimal media containing various combinations of supplements. (**B**) Closer magnification of the microcolonies formed in the *E. coli* mutants grown on M63 minimal media supplemented with glucose, vitamins, casamino acids, Tyrosine, Phenylalanine and without Tryptophan. Scale, 1 mm.

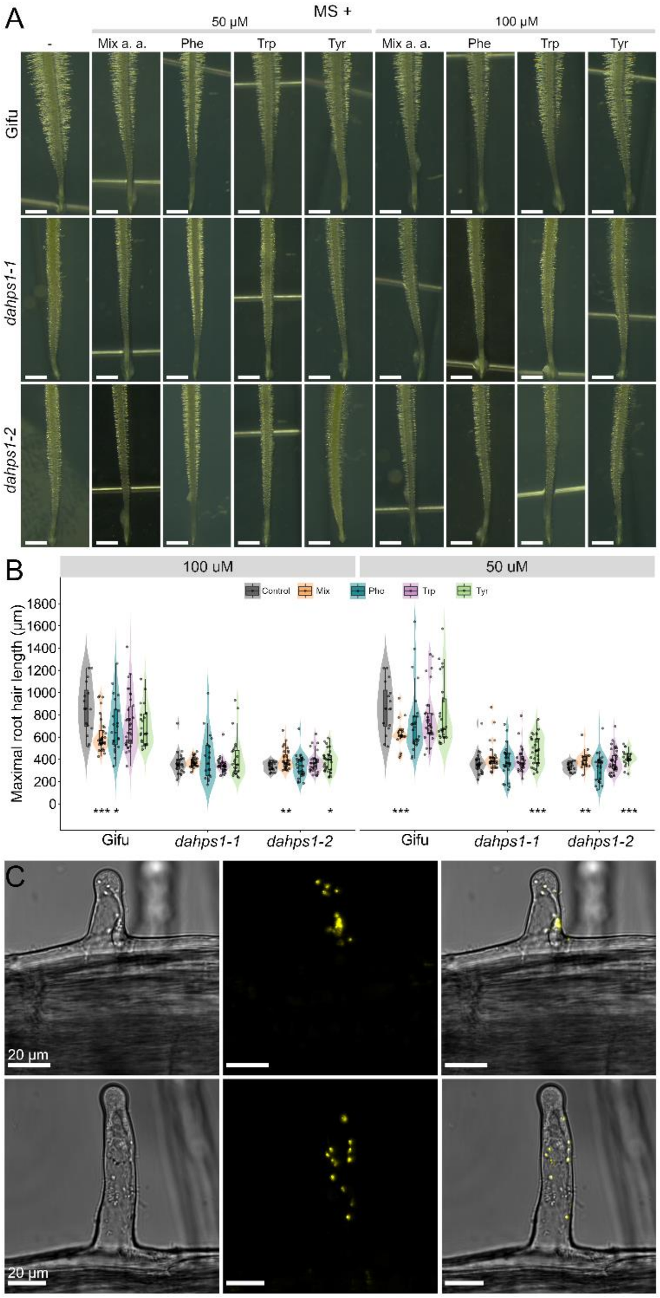

Supplemental Figure S6. Chemical complementation of *dahps1* mutants and subcellular localization of DAHPS1-YFP in *Lotus* transgenic roots. (**A**) Representative images of Gifu, *dahps1*-1, and *dahps1*-2 roots grown in MS medium supplemented individually with 50 or 100 µM of Phe, Trp, Tyr or a mixture of them. Scale, 1 mm (images captured at the same magnification). (**B**) Violin dot plots show the maximal root hair length recorded in Gifu, *dahps1*-1 and *dahps1*-2 grown in MS medium supplemented with various concentrations of Phe, Trp and Tyr. Violin boxplots: centre line, median; box limits, upper and lower quartiles; whiskers, 1.5× interquartile range; points, individual data points. The number of plants tested are indicated in Supplemental Figure 4B. Student's t-test of root hair length between Gifu and the two *dahps1* mutant alleles. *$p < 0.05$; **$p < 0.01$; ***$p < 0.001$. (**C**) Confocal microscopy images show the localization of DAHPS1-YFP in growing root hairs of *dahps1*-1 transgenic roots. Left panel, transmitted light; Middle panel, yellow fluorescence; Right panel, merged images.

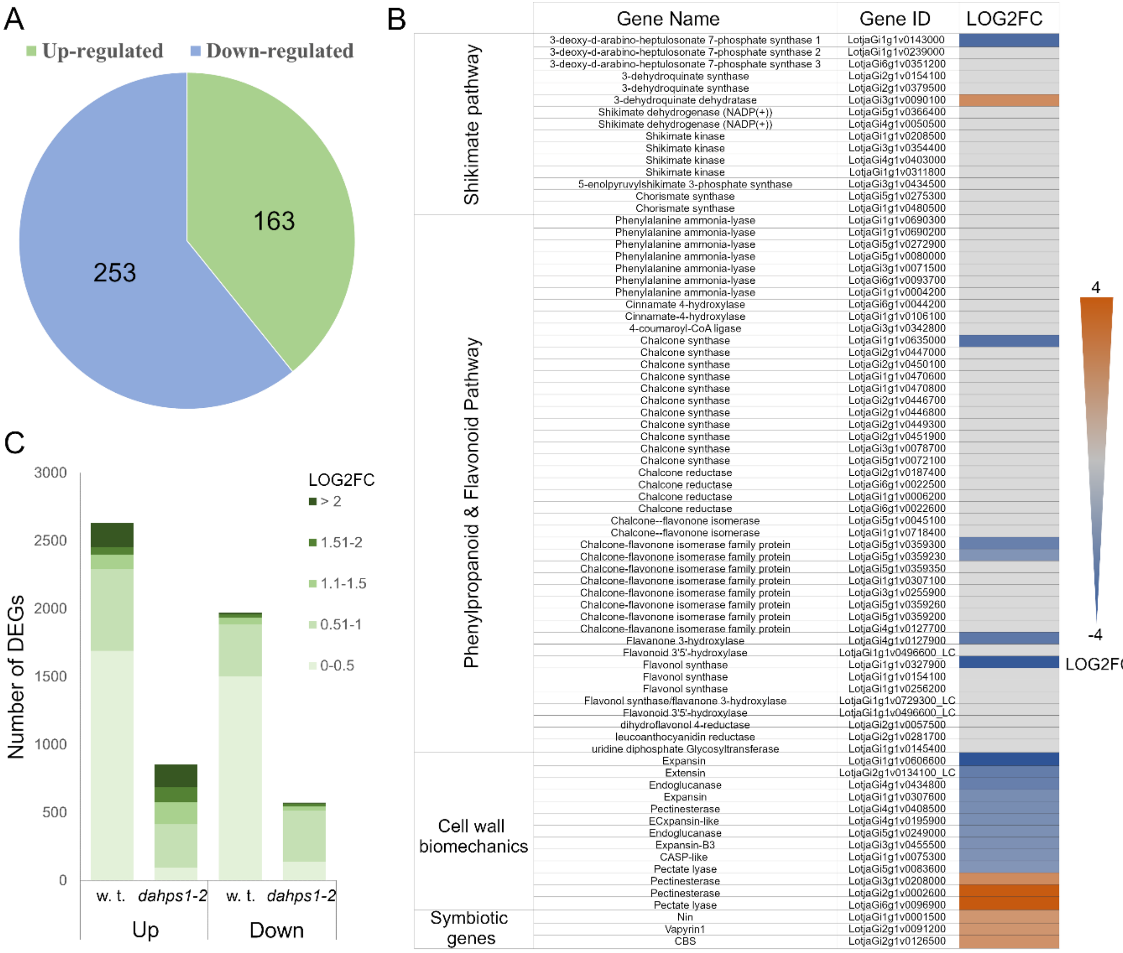

Supplemental Figure S7. DEG in uninoculated 5 days old roots of *dahps1*-2 mutant. (**A**) Total number of up/downregulated genes (p-adjust < 0.5, Log2FC ≥2) in *dahps1*-2 roots respect to uninoculated roots of similar age in Gifu. (**B**) Heatmap expression of genes associated to the shikimate, phenylpropanoid and flavonoid pathway in uninoculated *dahps1*-2 roots, relative to Gifu. DEG encoding cell wall proteins, symbiotic genes and *DAHPS1* are also included. (**C**) Number of DEG in Gifu and *dahps1*-2 roots at 5 dpi with IRBG74 (p-adjust < 0.5).

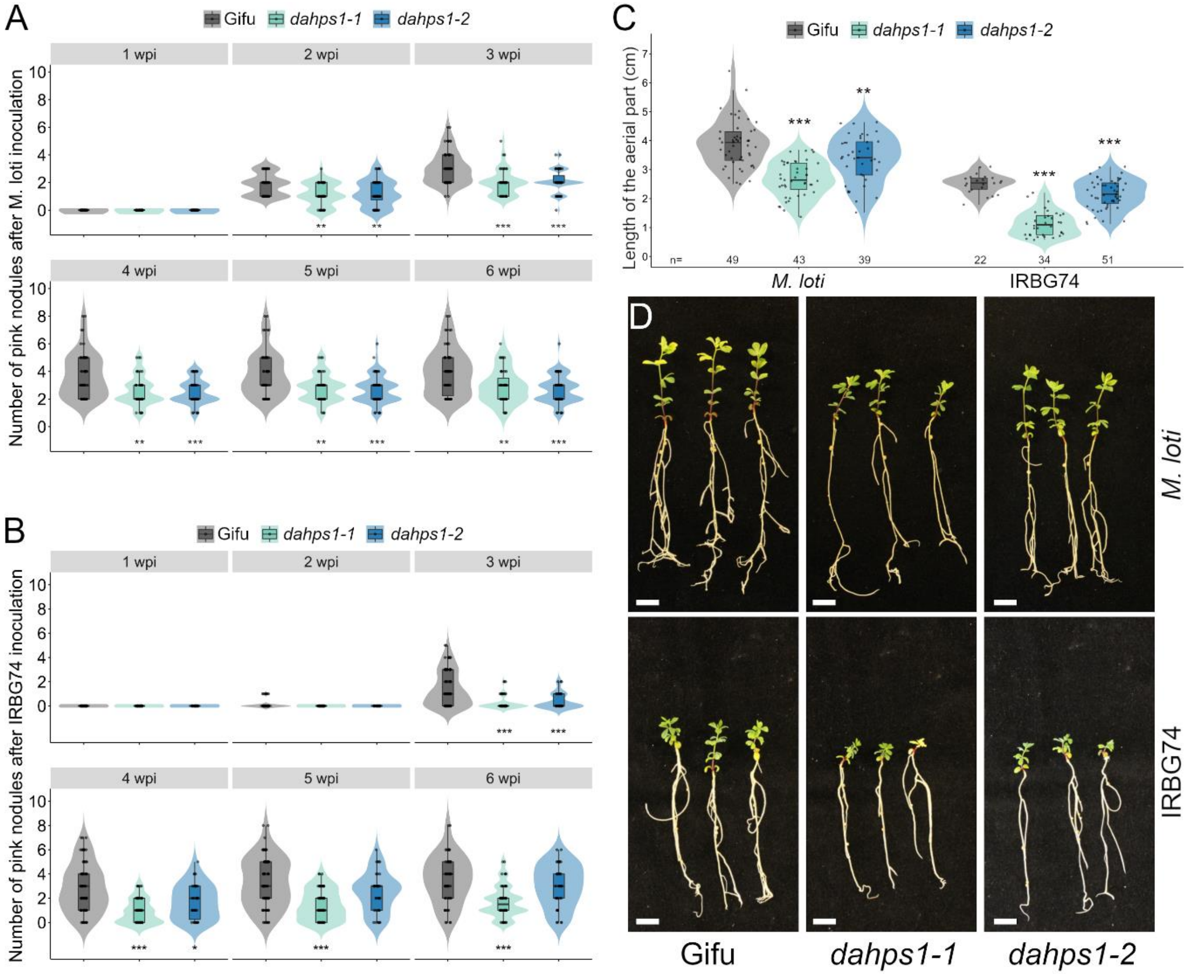

Supplemental Figure S8. Nodule formation and plant growth in the *dahps1*-1 and *dahps1*-2 mutants. Violin dot plots show the number of pink nodules recorded on Gifu (n ≥ 29), *dahps1*-1 (n ≥ 43) and *dahps1*-2 (n ≥ 29) at 1-6 wpi with *M. loti* (**A**) and IRBG74 (**B**). Mann–Whitney U-test of pink nodules (asterisks below the violin graphs indicates significant difference: *$p < 0.05$; **$p < 0.01$; ***$p < 0.001$) between Gifu and mutant plants. Length of the aerial part (**C**) and representative images of Gifu, *dahps1*-1 and *dahps1*-2 plants (**D**) at 6 wpi with *M. loti* and IRBG74. Scale, 1 cm. Student's t test of length of the aerial part between Gifu and the two *DAHPS1* mutant alleles inoculated with *M. loti* or IRBG74. **$p < 0.01$; ***$p < 0.001$. Violin boxplots: center line, median; box limits, upper and lower quartiles; whiskers, 1.5× interquartile range; points, individual data points.
